# Pansclerotic morphea is characterized by IFN-**γ** responses priming dendritic cell fibroblast crosstalk to promote fibrosis

**DOI:** 10.1172/jci.insight.171307

**Published:** 2023-08-22

**Authors:** Enze Xing, Feiyang Ma, Rachael Wasikowski, Allison C. Billi, Mehrnaz Gharaee-Kermani, Jennifer Fox, Craig Dobry, Amanda Victory, Mrinal K. Sarkar, Xianying Xing, Olesya Plazyo, Henry W. Chen, Grant Barber, Heidi Jacobe, Pei-Suen Tsou, Robert L. Modlin, John Varga, J. Michelle Kahlenberg, Lam C. Tsoi, Johann E. Gudjonsson, Dinesh Khanna

**Affiliations:** 1 Department of Dermatology;; 2 Division of Rheumatology, Department of Internal Medicine; and; 3 Department of Computational Medicine and Bioinformatics, University of Michigan, Ann Arbor, Michigan, USA.; 4 Department of Dermatology, UT Southwestern Medical Center, Dallas, Texas, USA.; 5 Division of Dermatology, Department of Medicine, UCLA, Los Angeles, California, USA.; 6 University of Michigan SSc Program, Ann Arbor, Michigan, USA.; 7 Taubman Institute, Michigan Medicine, Ann Arbor, Michigan, USA.

**Keywords:** Dermatology, Inflammation, Cellular immune response, Fibrosis, Skin

## Abstract

Pansclerotic morphea (PSM) is a rare, devastating disease characterized by extensive soft tissue fibrosis, secondary contractions, and significant morbidity. PSM pathogenesis is unknown, and aggressive immunosuppressive treatments rarely slow disease progression. We aimed to characterize molecular mechanisms driving PSM and to identify therapeutically targetable pathways by performing single-cell and spatial RNA-Seq on 7 healthy controls and on lesional and nonlesional skin biopsies of a patient with PSM 12 months apart. We then validated our findings using immunostaining and in vitro approaches. Fibrotic skin was characterized by prominent type II IFN response, accompanied by infiltrating myeloid cells, B cells, and T cells, which were the main IFN-γ source. We identified unique *CXCL9^+^* fibroblasts enriched in PSM, characterized by increased chemokine expression, including *CXCL9*, *CXCL10*, and *CCL2*. *CXCL9^+^* fibroblasts were related to profibrotic *COL8A1*^+^ myofibroblasts, which had enriched TGF-β response. In vitro, TGF-β and IFN-γ synergistically increased *CXCL9* and *CXCL10* expression, contributing to the perpetuation of IFN-γ responses. Furthermore, cell-to-cell interaction analyses revealed cDC2B DCs as a key communication hub between *CXCL9*^+^ fibroblasts and *COL8A1*^+^ myofibroblasts. These results define PSM as an inflammation-driven condition centered on type II IFN responses. This work identified key pathogenic circuits between T cells, cDC2Bs, and myofibroblasts, and it suggests that JAK1/2 inhibition is a potential therapeutic option in PSM.

## Graphical abstract

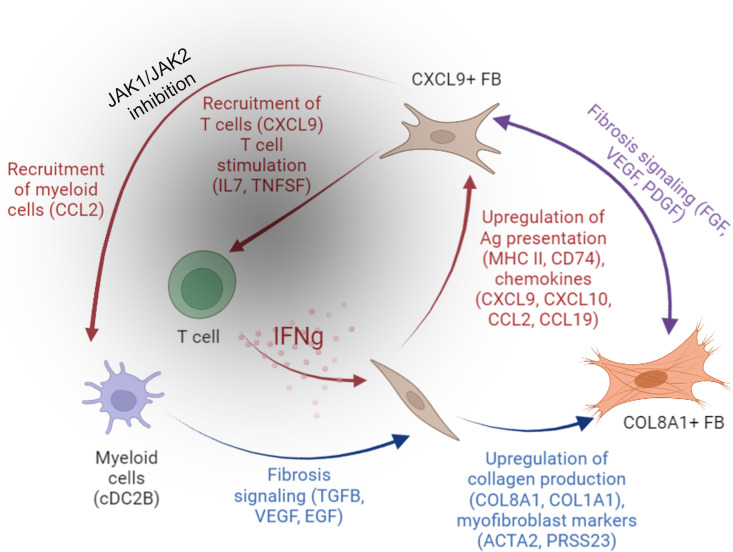

## Introduction

Pansclerotic morphea (PSM) is an extremely rare, difficult-to-treat disease, with a prevalence of less than 1 per 10,000,000 (1, 2). Limited knowledge exists on its pathogenesis, likely due to its rarity. PSM is clinically and epidemiologically distinct from other subtypes of morphea and systemic sclerosis (SSc), despite similarities in the skin manifestations of these diseases. Morphea occurs in 0.4–2.7 cases per 100,000 people (1, 3). As the rarest subtype of morphea, PSM is characterized by near-total body involvement of deeply sclerotic lesions. It is distinct from other subtypes of morphea due to extensive body involvement of sclerosis as well as the extension of lesions into s.c. fat and soft tissue, a phenotype that produces the associated morbidity (3, 4). However, despite its capacity to affect deeper s.c. tissues, fibrosis of the internal organs is not seen in patients with PSM, a clear differentiation from SSc (5). Furthermore, whereas SSc and morphea predominantly affect female patients, male patients are more likely to develop PSM (4, 6). These parallel but disparate presentations likely reflect differences in the immunological interplay underlying the pathogenesis of these diseases.

Many case reports have described the presentation, diagnosis, and potential therapeutic options for patients with PSM. However, there are no publications to date that describe the underlying disease mechanisms. Thus, this study sought to provide a detailed characterization of the cellular and molecular players underlying PSM’s pathogenesis using single-cell and spatial RNA-Seq approaches, and it helps identify therapeutically targetable pathogenic mechanisms, as well as to provide a resource for future investigations into this devastating disease.

## Results

### Single-cell RNA-Seq (scRNA-Seq) identifies diverse skin and immune cell populations from healthy controls and lesional and nonlesional skin from patients with PSM.

**Figure 1:**
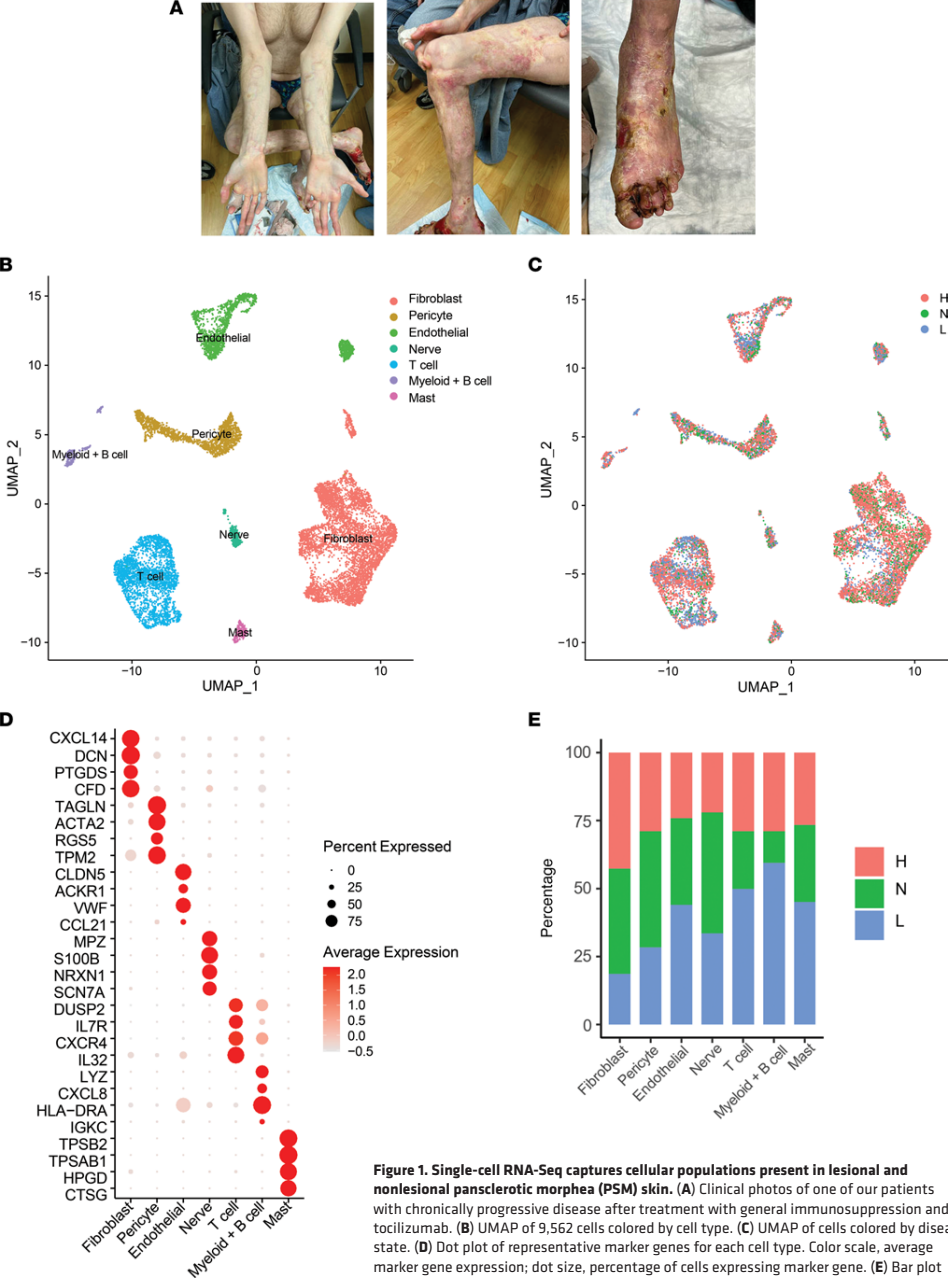
Single-cell RNA-Seq captures cellular populations present in lesional and nonlesional pansclerotic morphea (PSM) skin. (**A**) Clinical photos of one of our patients with chronically progressive disease after treatment with general immunosuppression and tocilizumab. (**B**) UMAP of 9,562 cells colored by cell type. (**C**) UMAP of cells colored by disease state. (**D**) Dot plot of representative marker genes for each cell type. Color scale, average marker gene expression; dot size, percentage of cells expressing marker gene. (**E**) Bar plot showing the relative contribution of the 3 disease states to the total number of each cell type. Values are normalized to the total number of cells for each disease state.

To compare the cellular composition and cell states of healthy control (HC) skin and lesional and nonlesional skin from patients with PSM, we performed scRNA-Seq from single-cell suspensions of biopsies from 7 HC donors and lesional and nonlesional skin from a single patient with PSM (Figure 1A) at 2 different time points, 12 months apart. The resulting single-cell data set comprised a total of 11,903 cells, with an average of 2,058 genes and 6,219 transcripts detected per cell. Visualization using Uniform Manifold Approximation and Projection (UMAP) revealed 19 distinct cell clusters (Supplemental Figure 1, A and B; supplemental material available online with this article; https://doi.org/10.1172/jci.insight.171307DS1) that were annotated as 7 distinct major cell types (Figure 1B), including fibroblasts (FBs), T cells, myeloid + B cells, mast cells, pericytes (Supplemental Figure 2), nerve cells, and endothelial cells (Supplemental Figure 3). Each major cell group contained cells from lesional, nonlesional, and HC skin (Figure 1, C and D). Notably, lesional T cells clustered conspicuously away from nonlesional tissue and HC, while other cell types displayed more overlapping patterns between HC and PSM skin. Cell composition analysis revealed increased proportions of inflammatory cells in lesional skin relative to nonlesional and HC skin, especially for the T cell and myeloid + B cell populations (Figure 1E).

### T cells from lesional and nonlesional PSM skin exhibit increased IFN-γ expression.

**Figure 2:**
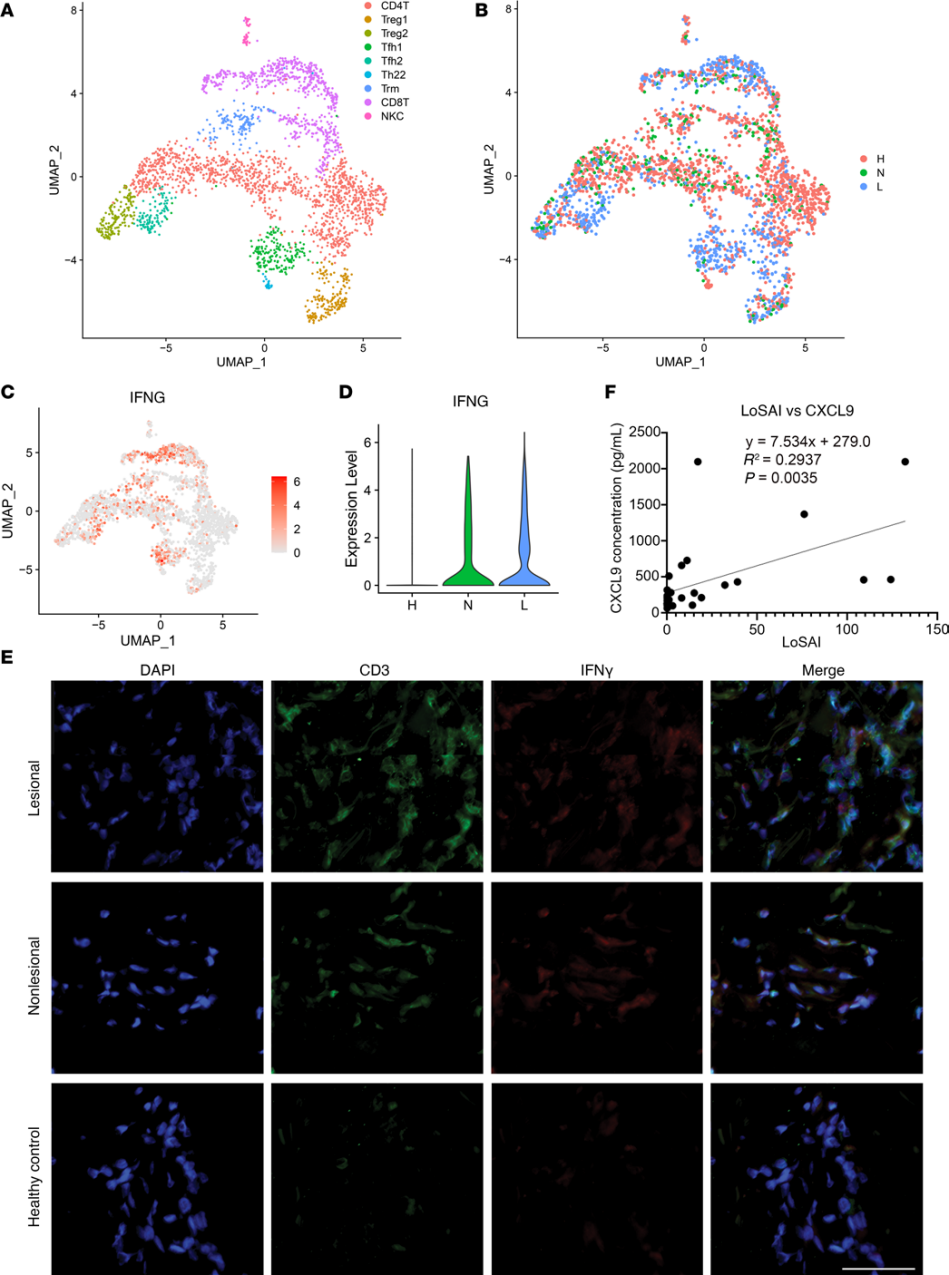
PSM exhibits high IFN-γ activity. (**A**) UMAP of 2,694 T cells colored by cell subtype. (**B**) UMAP of T cells colored by disease state. (**C**) Feature plot of IFNG expression by T cell subsets. (**D**) Violin plot of IFNG production by T cells separated by disease state. (**E**) Immunofluorescence of CD3 and IFN-γ in HC as well as lesional and nonlesional PSM. (**F**) Serum CXCL9 concentration plotted against disease severity (LoSAI) in 9 patients with PSM (linear regression analysis).

To understand the fundamental transcriptional differences driving the separation between T cells of varying disease states, we performed subclustering of the 2,694 T cells from our scRNA-Seq data set. We annotated the T cell subclusters into 9 subtypes, including CD4, CD8, 2 clusters of Tregs (Treg1, Treg2), 2 populations of Tfh cells (Tfh1, Tfh2), resident memory T cells (Trm), and NK cells (NKC) (Figure 2A and Supplemental Figure 4A). Tfh and Treg populations were especially enriched in PSM lesional skin compared with nonlesional and HC skin, while nonlesional skin showed increased proportions of CD8, Treg1, Treg2, and Trm compared with HC (Figure 2B and Supplemental Figure 4B).

To investigate T cell activity, we assessed their cytokine gene expression, which revealed prominent expression of *IFNG* across Tfh1, Tfh2, Trm, and CD8^+^ T cells, while other key T cell cytokines representing Th2 and Th17 polarization showed undetectable levels of expression (Figure 2C and Supplemental Figure 4C). Accordingly, violin plots revealed increased expression of *IFNG* in total T cells from both lesional and nonlesional PSM skin relative to HC (Figure 2D). Immunofluorescence (IF) staining identified enrichment of CD3^+^ cell clusters with intracellular IFN-γ in lesional and nonlesional skin, confirming that T cells are a major source of type II IFN in PSM skin (Figure 2E). Given the overlap in fibrosis between SSc and PSM, we then examined the difference in IFN-γ expression in SSc versus PSM skin. Staining of SSc lesional samples also revealed the presence of IFN-γ, while colocalization with CD3^+^ cells was not observed (Supplemental Figure 5).

To assess the level of IFN-γ responses in PSM, we determined serum concentrations of CXCL9, an IFN-γ–inducible chemokine, by ELISA in an independent cohort of 9 patients with PSM at several time points (7). We then performed correlation analysis comparing serum CXCL9 concentration at each time point to the patient’s Localized Scleroderma Assessment Tool (LoSCAT) activity index (LoSAI) score (Figure 2F). We found that serum levels of CXCL9 were positively associated with higher LoSAI scores (*r*^2^ = 0.2937, *P* = 0.0035), supporting the evidence that IFN-γ plays a critical role and promotes heightened disease activity in PSM.

### PSM FBs show an increased type II IFN signature and have increased expression of extracellular matrix genes.

**Figure 3:**
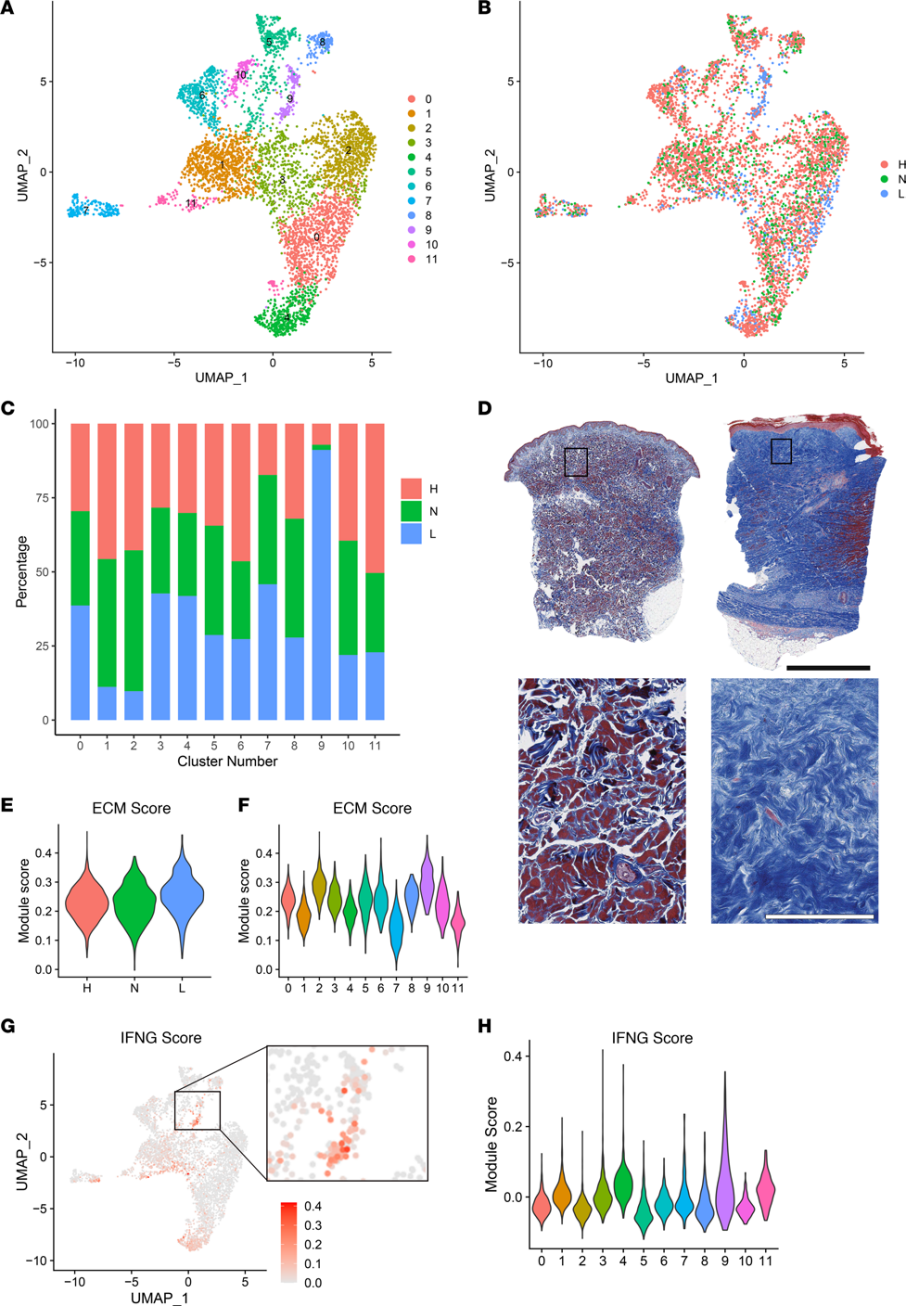
Lesional versus HC exhibit robust differences in transcriptomic profiling, and cluster 9 fibroblasts are the main lesional population contributing to ECM production. (**A**) UMAP of 5,152 fibroblasts colored by fibroblast subcluster. (**B**) UMAP of cells colored by disease state. (**C**) Bar plot showing the relative contribution of the 3 disease states to the total number of each fibroblast subcluster. Values are normalized to the total number of cells for each disease state. (**D**) Trichrome staining in HC and PSM. Black scale bar: 2 mm. White scale bar: 200 μm. (**E** and **F**) Violin plots of fibroblast scores for ECM production split by disease state (**E**) and by fibroblast subset (**F**). (**G**) Feature plots of module scores for IFN-γ. (**H**) Violin plots of fibroblast scores for the indicated cytokine modules split by fibroblast subcluster.

FBs play a central role in promoting fibrosis in diseases such as morphea and SSc (8, 9). To elucidate the differences between FBs in PSM compared with HC skin, we performed subclustering on the 5,152 FBs in our data set, revealing 11 subclusters (Figure 3A and Supplemental Figure 6A). Clusters 1, 2, 10, and 11 were decreased in lesional skin compared with nonlesional and HC, while clusters 0, 3, 4, 7, and 9 were enriched, with cluster 9 having the highest proportion in lesional PSM skin (Figure 3, B and C). Trichrome staining of PSM biopsies showed dense extracellular collagen deposition (Figure 3D).

To examine the capacity for ECM production by PSM FBs, we calculated an ECM module score across each FB cluster. As expected, lesional PSM FBs had a higher ECM score compared with both nonlesional and HC FBs, with cluster 9 FBs having the highest ECM score (Figure 3, E and F). Together, these data illustrate increased ECM production in PSM skin and identify cluster 9 FBs as likely key drivers of the fibrotic process.

To better understand the activation of cluster 9 FBs, we performed GO analysis on significantly differentially expressed genes (DEGs) between lesional and HC cluster 9 FBs (FDR < 0.05). PSM cluster 9 FBs showed heightened type II IFN responses (Supplemental Figure 6B). In addition, cluster 9 FBs showed enrichment for genes involved in the antigen presentation pathway, which has been previously reported in FBs secondary to IFN-γ stimulation (10). To confirm the specificity of PSM FB IFN-γ response (Figure 3G), we compared their gene expression against bulk RNA-Seq data from IFN-γ–stimulated cultured dermal FBs (Figure 3H). Notably, only half of the cluster 9 FBs had an IFN-γ signal, indicating a transcriptionally distinct subcluster within FB cluster 9.

### PSM-specific FBs constitute 2 subpopulations of FBs associated with leukocyte recruitment machinery in response to IFN-γ.

**Figure 4:**
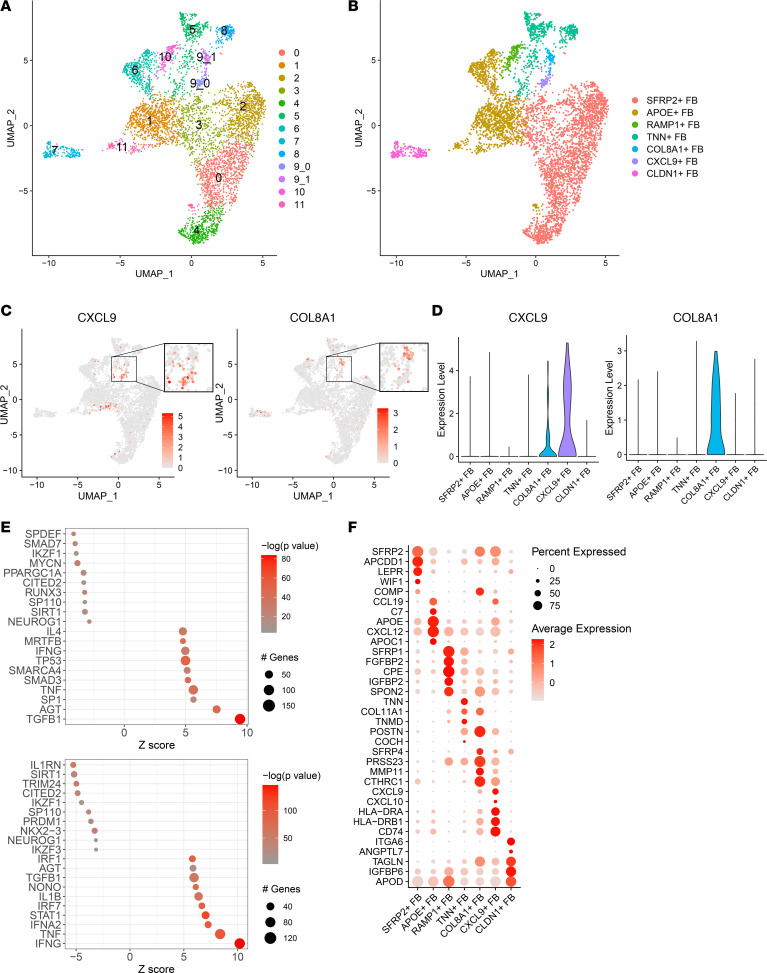
Cluster 9 fibroblasts are broken up into proinflammatory and profibrotic subgroups. (**A**) UMAP of fibroblasts with subcluster 9 broken into 9_0 and 9_1. (**B**) Annotated UMAP, with fibroblasts colored by subtype. (**C**) Feature plot of *CXCL9* and *COL8A1* gene expression. (**D**) Violin plot of *CXCL9* and *COL8A1* gene expression. (**E**) The upstream regulator analysis of *CXCL9^+^* and *COL8A1*^+^ FB marker genes. (**F**) Dot plot of representative marker genes for each fibroblast subtype.

We therefore manually separated cluster 9 into clusters 9_0 and 9_1 (Figure 4A). Annotation of these subclusters based on published FB marker genes revealed 7 FB subtypes — *SFRP2*^+^, *APOE*^+^, *RAMP1*^+^, *TNN*^+^, *CLDN1*^+^, *CXCL9^+^*, and *COL8A1*^+^ FBs — with the latter 2 representing clusters 9_0 and 9_1, respectively (Figure 4B). Feature plots depicting *CXCL9* and *COL8A1* gene expression reveal *CXCL9^+^* FBs map onto the same location as IFN-γ responsive FBs (9_0), whereas *COL8A1*^+^ FBs represent the second subset of cluster 9 (9_1) (Figure 4C). Violin plots confirm that *CXCL9* is expressed most robustly by *CXCL9^+^* FB, while *COL8A1* is expressed almost exclusively by *COL8A1*^+^ FB (Figure 4D).

To determine the differential regulation between *CXCL9^+^* and *COL8A1*^+^ FBs, we identified significant cluster marker genes (FDR < 0.05) and used Ingenuity Pathway Analysis (IPA) to identify their upstream regulators. Inflammatory cytokines, including IFN-γ, TNF, and IFNA2, were the top upstream regulators in *CXCL9^+^* FBs, whereas *COL8A1*^+^ FBs had profibrotic TGF-β and SMAD3 as the key upstream regulators (Figure 4E). TGF-β was predicted as a less significant upstream regulator of *CXCL9^+^* FBs compared with cytokines IFN-γ, TNF, and IFNA2 based on *z* score, and TNF and IFN-γ were likewise predicted to be less probable upstream regulators for *COL8A1*^+^ FBs.

**Figure 5:**
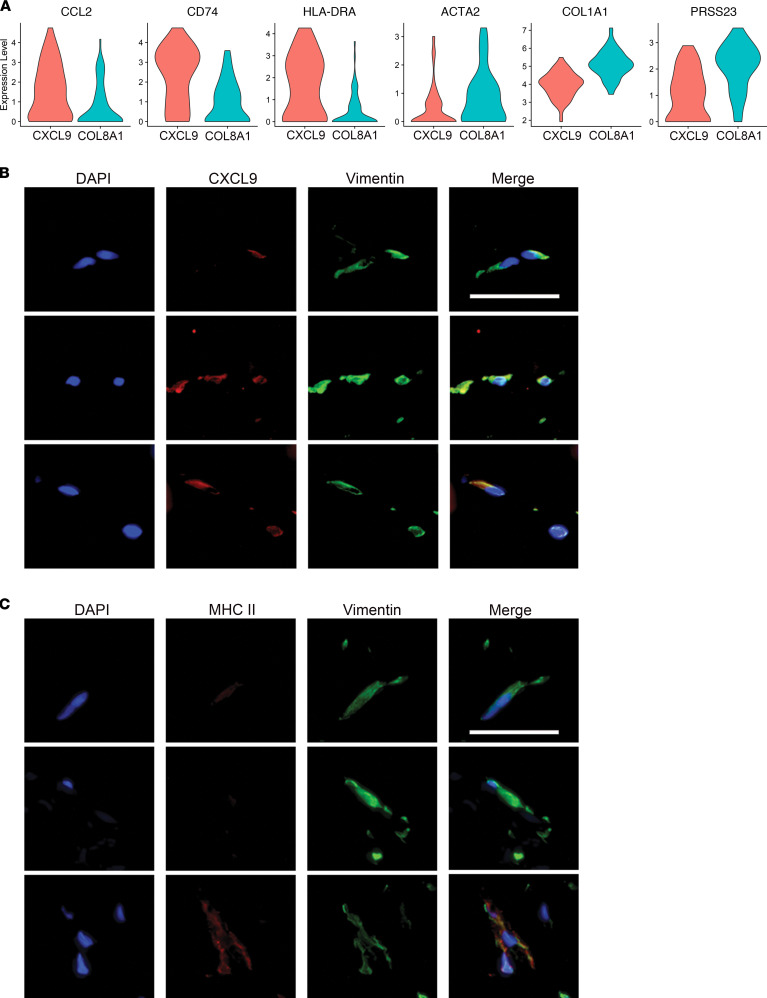
CXCL9^+^ FBs are IFN-γ responsive and distinct from myofibroblasts. (**A**) Violin plot of IFN-γ response and myofibroblast marker genes in *CXCL9^+^* and *COL8A1*^+^ FBs. (**B** and **C**) Immunofluorescence of *CXCL9* and vimentin (**B**) or vimentin and MHC II (**C**) in HC as well as lesional and nonlesional PSM skin.

GO analysis of DEGs between *CXCL9^+^* and *COL8A1*^+^ FBs suggested that *CXCL9^+^* FBs are active participants in inflammation, including antigen presentation and cellular response to proinflammatory cytokines IFN-γ, type I IFNs, and TNF. *COL8A1*^+^ FB DEGs were enriched for processes involved in profibrotic responses (Figure 4F and Supplemental Figure 7A). Violin plots reiterate increased expression of myofibroblast markers *ACTA2*, *COL1A1*, and *PRSS23* in *COL8A1*^+^ FBs, indicating myofibroblast differentiation and profibrotic activity, while *CXCL9^+^* FBs have upregulation of *CD74*, *HLA-DRA*, and *CCL2* (Figure 5A) (9, 11). IF confirmed the presence of *CXCL9*^+^vimentin^+^ and MHC II^+^vimentin^+^ FBs present in PSM skin (Figure 5, B and C). Staining was also performed on SSc and morphea skin due to similar presentations of cutaneous fibrosis. In contrast, no double-positive FBs were present in morphea, and only MHC II^+^vimentin^+^ FBs were identified in SSc, solidifying the *CXCL9^+^* FB subset as a PSM-specific inflammatory FB population (Supplemental Figure 7, B and C).

**Figure 6:**
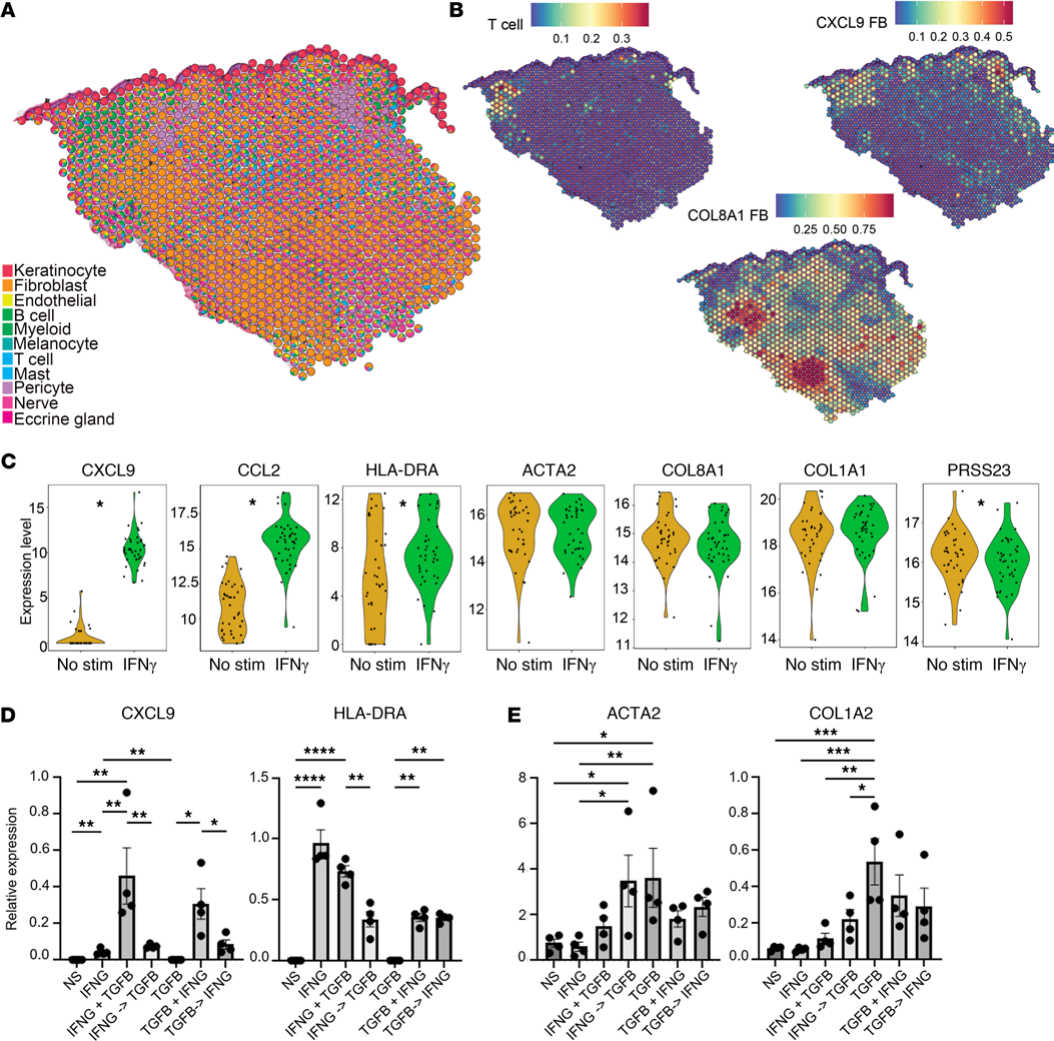
Spatial transcriptomics shows spatial proximity of CXCL9^+^ and COL8A1^+^ and of IFN-γ and TGF-β synergy in inducing CXCL9 expression in fibroblasts. (**A**) Spatial plot showing deconvoluted cell types overlaid on lesional PSM H&E. Coordinates of the spot correspond to the location in the tissue. (**B**) Subtype deconvolution describes locations of T cells and *CXCL9^+^* and *COL8A1^+^* fibroblasts on lesional PSM slice. (**C**) Bulk RNA-Seq data of healthy fibroblasts after 6-hour IFN-γ stimulation. (**D** and **E**) Relative expression of IFN-γ response genes (**D**) or myofibroblast markers (**E**) after 72-hour priming with IFN-γ or TGF-β, followed by another 72-hour incubation with the same (IFN-γ, TGF-β), other (IFN-γ**→**TGF-β, TGF-β**→**IFN-γ), or both (IFN-γ+TGF-β, TGF-β+IFN-γ) cytokines (1-way ANOVA with Sidak test, 2-tailed unpaired *t* test, *n* = 4). *P ≤ 0.05.

Spatial sequencing was performed using the 10× Visium platform to provide insight into the localization of the fibrotic process and its relation to FB subsets. Conditional autoregressive-based deconvolution method using the cell-type markers from the single-cell analyses was performed for each spot of the spatial-sequencing grid and displayed as a scatter-pie plot superimposed on the H&E staining of the matching tissue (Figure 6A). Consistent with the histology of the tissue, transcriptomic signatures of keratinocytes localized to the epidermis, while signatures for B cells, myeloid cells, and T cells were scattered in small foci throughout the dermis. FB signatures were found to be robustly distributed throughout the dermis. In contrast, nonlesional skin exhibited similar distribution of FBs but maintained a more uniform distribution of immune, nerve, and eccrine gland cells throughout the skin (Supplemental Figure 8A).

We utilized cell subtype marker genes from our scRNA-Seq data set to create heatmaps that identify the locations of T cells and FB subsets as well as expression levels of *CD3D*, *CXCL9*, *CD74*, *HLA-DRA*, *ACTA2*, *COL8A1*, and *COL1A1* in lesional PSM skin (Figure 6B and Supplemental Figure 8B). T cells were located primarily in the superficial dermis and aligned with *CD3D* expression. *CXCL9^+^* FBs correlated with T cell location in lesional skin and colocalized with HLA-DRA and CD74 expression. Finally, *CXCL9*^+^ FBs were clustered together in the upper dermis, while *COL8A1*^+^ FBs were in an adjacent cluster in the lower dermis, aligning with *COL8A1* and *ACTA2* expression. Robust *COL1A1* expression was observed throughout both superficial and deep dermis in lesional PSM skin. Thus, we were able to identify *CXCL9*^+^ FBs and *COL8A1*^+^ FBs as spatially related FB subsets, with the *CXCL9^+^* subset situated closer to the T cell source of IFN-γ.

To address the role of IFN-γ on FB function, we assessed the mRNA expression of *CXCL9*, *CCL2*, *HLA-DRA*, *ACTA2*, *COL8A1*, *COL1A1*, and *PRSS23* by HC FBs after IFN-γ and TGF-β stimulation. IFN-γ treatment resulted in significant upregulation of *CXCL9*, *CCL2*, and *HLA-DRA*. Notably, IFN-γ treatment did not significantly alter the expression of collagen genes (Figure 6C). In contrast, TGF-β stimulation increased *COL1A1*, *CXCL9*, and *CCL2* mRNA expression (Supplemental Figure 7D). *CXCL9^+^* and *COL8A1*^+^ FB populations displayed both IFN-γ and TGF-β as potential upstream regulators.

To determine if IFN-γ and TGF-β act synergistically under these circumstances, we primed primary dermal FBs from HCs for 72 hours with either IFN-γ or TGF-β, followed by restimulation with the same, the other, or both cytokines for another 72 hours. *CXCL9*, *CXCL10*, *CD74*, and *HLA-DRA* had increased mRNA expression after IFN-γ treatment compared with unstimulated FBs, as determined by quantitative PCR (qPCR) (Figure 6D and Supplemental Figure 8C). FBs primed with IFN-γ synergized with TGF-β to induce the expression of *CXCL9* and *CXCL10*. In contrast, *HLA-DRA* and *CD74* expression levels were dependent on IFN-γ stimulation, with TGF-β stimulation not further affecting their expression. Conversely, *ACTA2*, *COL1A1*, and *COL8A1* were significantly increased with TGF-β stimulation and did not further increase with subsequent IFN-γ (Figure 6E).

### Robust infiltration of myeloid and B cells in lesional PSM skin.

**Figure 7:**
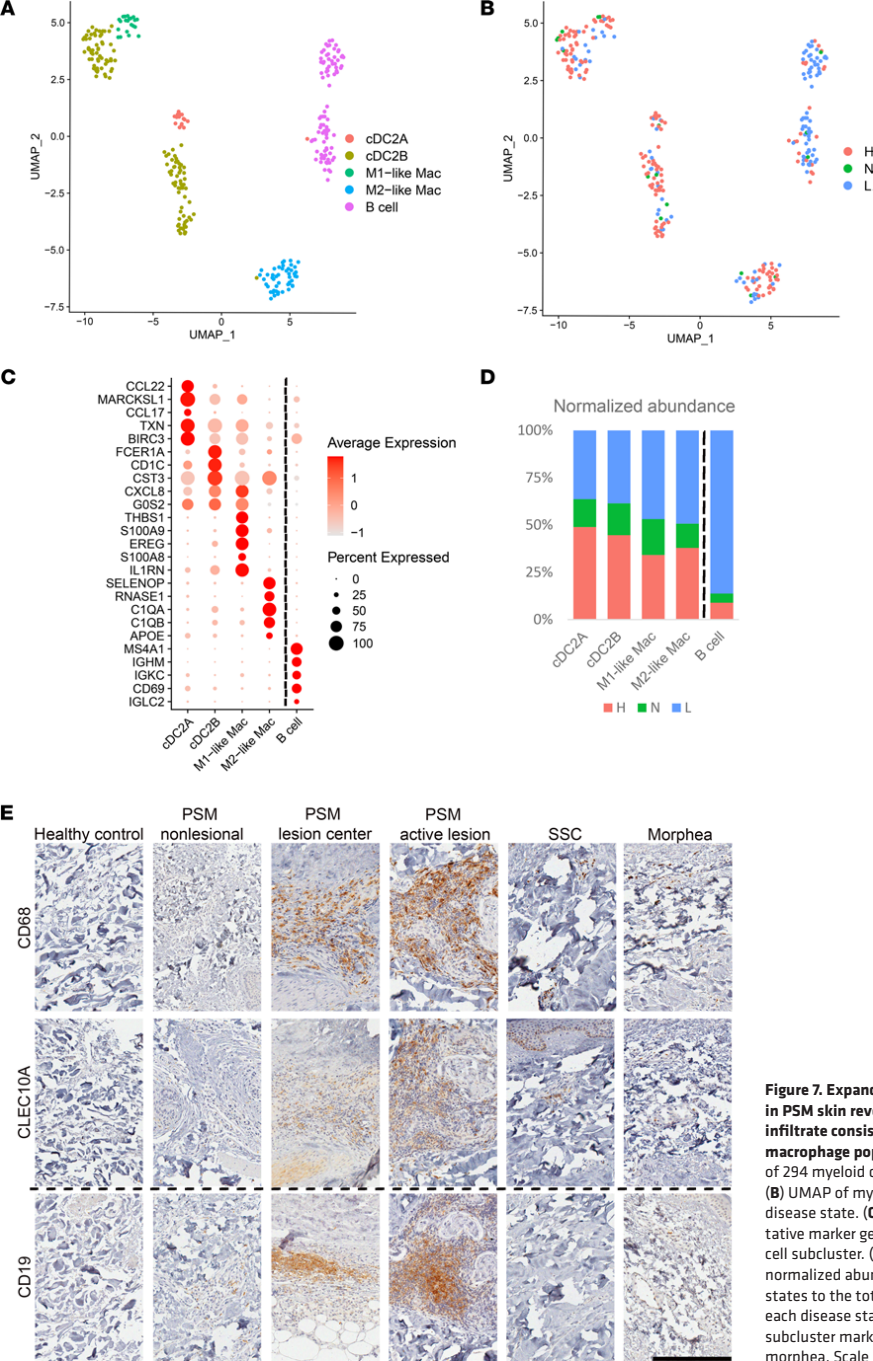
Expanded myeloid population in PSM skin reveals inflammatory infiltrate consisting of B cells, cDCs, and macrophage populations. (**A**) UMAP of 294 myeloid colored by cell subtype. (**B**) UMAP of myeloid cells colored by disease state. (**C**) Dot plot of representative marker genes for each myeloid cell subcluster. (**D**) Bar plot showing the normalized abundance of the 3 disease states to the total number of cells in each disease state. (**E**) IHC for myeloid subcluster markers in HC, PSM, SSc, and morphea. Scale bar: 200 μm.

We next analyzed the presence of myeloid and B cells in PSM skin. Subclustering of 294 cells revealed 5 distinct cell types, including cDC2A, cDC2B, M1-like macrophages, M2-like macrophages, and B cells (Figure 7, A–D). These cell populations were validated by IHC staining and contrasted against morphea, SSc, and HC skin (Figure 7E and Supplemental Figure 9). Notably, while myeloid and B cell populations were present in SSc and morphea lesions, the level of infiltration was far less than what we observed in PSM skin. To determine functional differences in myeloid function, we identified DEGs between the various myeloid subtypes in lesional PSM versus HC skin. cDC2B exhibited the highest number of DEGs, while cDC2A and M1-like macrophages had no identifiable DEGs (Supplemental Figure 10A). Upstream regulator analysis of cDC2B DEGs revealed IFN-γ as the most significant upstream regulator (Supplemental Figure 10B). GO analysis of lesional cDC2Bs and M2-like macrophage DEGs demonstrated enrichment for cellular responses to both type II and type I IFNs as well as enrichment for genes involved in antigen processing and presentation (Supplemental Figure 10C).

### T cell–cDC2B–myofibroblast crosstalk links immune cells with profibrotic FB subsets.

**Figure 8:**
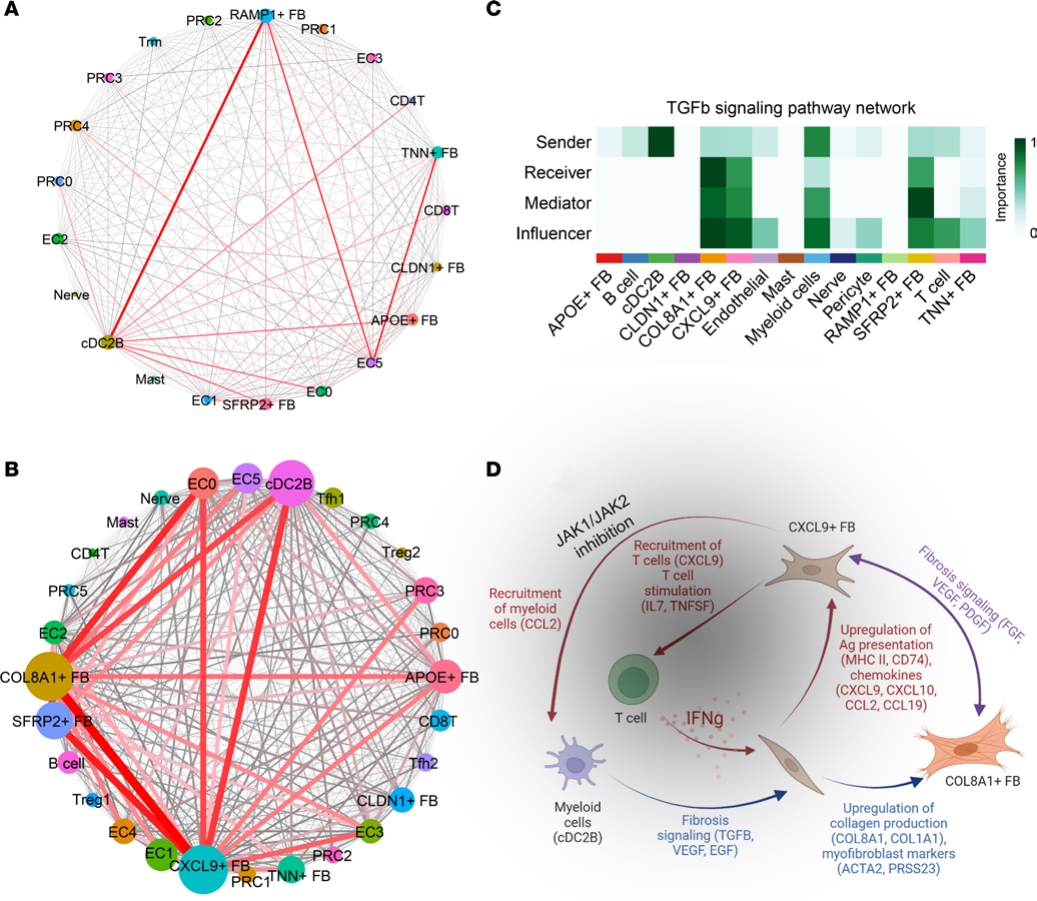
Cell-to-cell interactions in lesional skin promote the development of a profibrotic loop involving T cells, cDC2Bs, and FBs. (**A** and **B**) Connectome web analysis of interacting cell types. Line thickness is proportional to the number of interactions between 2 nodes, and cell type node size is proportional to the number of interactions to and from the cell type. (**C**) Heatmap showing the relative importance of each cell group based on the computed network centrality measures of the TGF-β signaling network. (**D**) Hypothesized T cell/FB/cDC2B crosstalk. Red indicates proinflammatory signaling, blue indicates profibrotic signaling, and purple indicates intra-FB interactions. Gray overlay indicates the area of disruption in the circuit from JAK1/JAK2 inhibition.

To understand the cell-to-cell interactions occurring in PSM, we performed ligand-receptor (L-R) analysis among major cell types in our data set using CellPhoneDB (12). To assess for changes between PSM lesional, nonlesional, and healthy skin, each L-R pair was assigned to the disease state in which it had the highest interaction score. Plotting these interactions revealed predicted shifts from normal to nonlesional to lesional PSM skin. Homeostatic interactions in healthy skin centered around cDC2B interactions with *RAMP*^+^ FBs as well as other cell subtypes (Figure 8A). *RAMP*^+^ FBs continue to be active in nonlesional PSM skin, contacting *TNN*^+^ and *SFRP2*^+^ FBs and several endothelial subpopulations (Supplemental Figure 11A). However, in lesional PSM skin, cellular interactions shifted toward *COL8A1*^+^ and *CXCL9^+^* FBs, with the strongest cell-to-cell interactions occurring between these 2 FB subsets, including *FGF2*, *FGF7*, *PDGFD*, *TGFB3*, and *VEGFB*. Other cell types — including *SFRP1*^+^ FBs, cDC2B, and endothelial cells — may contribute to this crosstalk (Figure 8B).

**Figure 9:**
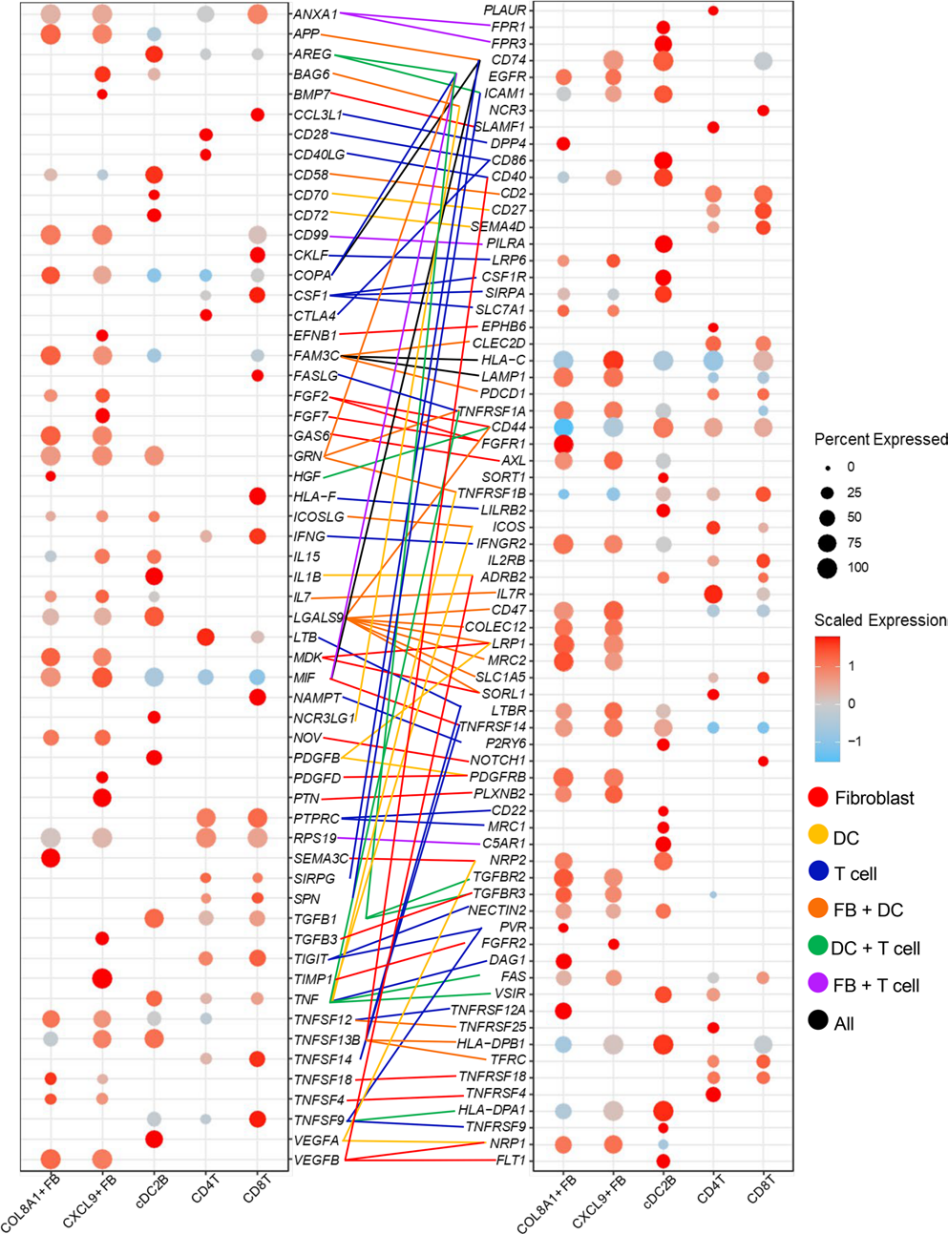
Predicted L-R interactions in lesional PSM skin. Dot plots show the expression of ligands (left) and receptors (right) in *COL8A1*^+^ FBs, *CXCL9^+^* FBs, cDC2Bs, and T cells. Lines represent the L-R interactions present, and the line color indicates the ligand-providing cell type.

Visualization via heatmap revealed *CXCL9^+^* FBs, *COL8A1*^+^ FBs, *APOE*^+^ FBs, EC0, and cDC2Bs as the most frequent ligand expressors in lesional interactions, and *CXCL9^+^* FBs, *COL8A1^+^* FBs, cDC2Bs, *SFRP2*^+^ FBs, and *APOE*^+^ FBs as the most frequent receptor expressors (Supplemental Figure 11B). L-R interactions revealed that FBs may contribute to T cell stimulation via *IL7*, *TNFSF4*, *TNFSF12*, *TNFSF13B*, and *TNFSF18* (Figure 9). *CXCL9*^+^ and *COL8A1*^+^ FBs may also mutually promote fibrosis through *FGF*, *PDGF*, and *VEGF*. We also utilized CellChat to better visualize functionally related fibrotic pathways between different cell subtypes (13). This showed cDC2Bs as a major predicted source of *TGFB* (Figure 8C and Supplemental Figure 11C), *VEGF* (Supplemental Figure 11D), and *EGF* (Supplemental Figure 11E) signaling in PSM lesional skin. TGF-β signaling was received predominantly by *COL8A1*^+^ FBs, followed by *CXCL9^+^* and *SFRP2*^+^ FBs. EGF signaling was observed in *CLDN1*^+^, *COL8A1*^+^, *CXCL9^+^*, and *SFRP2*^+^ FB populations, whereas VEGF primarily targeted endothelial cell populations. Overall, these data illustrate an interactive profibrotic and proinflammatory circuit between T cells, cDC2Bs, and FBs (Figure 8D).

## Discussion

We provide the first report to our knowledge to comprehensively characterize the cellular composition and molecular interactions in lesional skin in PSM. Our findings highlight a key role for T cell–derived IFN-γ as the most pronounced inflammatory signal present in PSM skin and as a potential primer of the profibrotic responses enriched in PSM skin. We describe a positive feedback and amplification circuit where IFN-γ stimulation of FBs results in upregulation of the chemokines *CXCL9* and *CXCL10* in FBs, as reflected by the enriched *CXCL9^+^* FB subset that was observed in PSM skin, which in turn may promote an increased influx of T cells, thereby sustaining and amplifying an environment dominated by prominent type II IFN responses. Through activation of *CXCL9^+^* FBs and recruitment of cDC2B DCs via CCR2, which produce profibrotic ligands including TGF-β, this circuit establishes profibrotic responses through activation of *COL8A1*^+^ myofibroblasts and deposition of type I collagen and other extracellular matrix components (14).

Our results suggest that PSM is an inflammatory disease that shares some pathogenic features with morphea and SSc (1, 5). However, there are notable differences among these fibrosing skin diseases. While classic morphea has a prominent Th1/IFN-γ signature, with elevated CXCL9, CXCL10, and IFN-γ^+^ T cells in the blood and skin of patients with active disease, there is no reported evidence of an increase in TGF-β activity (15–17). In SSc, vasculopathy and characteristic endothelial changes are prominent, along with inflammatory response skewed toward Th2 responses, IL-6, and TGF-β — features that we do not observe in PSM skin — suggesting that, despite shared profibrotic responses, these 3 diseases are largely distinct (18–20).

A notable finding in our study was the prominent type II IFN response in PSM skin, but IFN-γ is generally regarded as an antifibrotic cytokine in multiple organ systems. Both animal and human studies demonstrate that IFN-γ treatment reduces fibrosis due to suppression of TGF-β responses and decreased expression of ECM components (21–24). However, there is evidence to indicate that IFN-γ priming may also promote fibrosis, as shown in murine models of pulmonary fibrosis where IFN-γ KO or T cell depletion resulted in decreased fibrosis (25–27). Similarly, in classic morphea, dermal fibrosis has recently been linked to FB expression of CXCL9 (28). Consistent with these findings, our in vitro data show that IFN-γ did not directly induce the expression of collagen or myofibroblast markers in FBs.

IFN-γ stimulation induces expression of MHC II in FBs in vitro — a feature of the *CXCL9*^+^ FB population that is highly enriched in PSM skin. FBs have previously been reported to present antigens to T cells via IFN-γ–induced MHC II (10). This suggests that IFN-γ may mediate antigen presentation capabilities in FBs. However, this scenario would need to be addressed in future studies. Considering the role of TGF-β in myofibroblast differentiation and fibrosis (29), it is noteworthy that our in vitro data provide evidence for synergism between TGF-β and IFN-γ in driving *CXCL9* and *CXCL10* expression, with IFN-γ only partially suppressing the profibrotic role of TGF-β, thereby hinting at a complex interplay between these 2 cytokines in PSM skin.

Another notable finding is the identification in PSM lesional skin of proinflammatory *CXCL9^+^* FBs that have high expression of multiple proinflammatory cytokines and chemokines, including *IL7*, *TNFSF4*, *TNFSF12*, *TNFSF13*, *TNFSF18*, *CXCL9*, *CXCL10*, and *CCL2*. Proinflammatory FB populations have been recently described in other inflammatory skin diseases, including Th2 responsive *COL6A5^+^COL18A1^+^* FBs in atopic dermatitis and T cell–attracting CXCL9/CXCL10-expressing FBs in vitiligo (30, 31). However, neither atopic dermatitis nor vitiligo is associated with fibrosis.

Using spatial-Seq data, we demonstrated that T cells and myeloid cells corresponded with the locations of *CXCL9^+^* FBs. These myeloid cells had marker gene expression consistent with cDC2Bs, which had prominent TGF-β expression (32). *COL8A1^+^* myofibroblasts, defined by the expression of *ACTA2* (encoding α-smooth muscle actin), *PRSS23*, and elevated *COL1A1* transcription, were the most prominent TGF-β receiver in PSM skin by L-R analysis (9, 11). TGF-β is well known as a key regulator in FB-to-myofibroblast transition, promoting proliferation, survival, and ECM production (33, 34). While no reports exist to our knowledge on myofibroblasts in PSM skin, myofibroblasts have been identified in morphea and in SSc (9, 35). TGF-β may also act on and promote the generation of *CXCL9^+^* FBs and, as shown in our in vitro data, may synergize with IFN-γ to promote expression of the Th1/Tc1 chemokines CXCL9 and CXCL10, revealing a secondary mechanism by which TGF-β promotes and maintains type II IFN responses in PSM skin. This amplification circuit may explain the increased likelihood of PSM to be chronic and progressive compared with morphea, where IFN-γ–mediated inflammation is present but elevations in TGF-β response are not (15).

Finally, our study shows enrichment of both Tfh and B cell populations in PSM lesional skin. This is particularly noteworthy, as approximately one-third of patients with PSM have autoantibodies; however, the clinical relevance of these autoantibodies remains unclear. Together, these data suggest the potential for tertiary lymphoid organ development in PSM skin. While we did not identify the presence of germinal B cell, or plasma cell, markers in our B cell population, this would be an important avenue for future exploration.

Overall, our data support IFN-γ as a central inflammatory mediator upstream of fibrosis in PSM skin. This is paralleled in a recent report that identified gain-of-function mutations in STAT4, a known inducer of IFN-γ production and response, in 4 patients with a familial inheritance of PSM (7, 36). Since IFN-γ signals through JAK1/JAK2 and downstream STAT1 phosphorylation, nuclear translocation, and target gene transcription, our data suggest that this signaling pathway may be a potential therapeutic target for PSM, as recently demonstrated, where oral ruxolitinib, a JAK1/JAK2 inhibitor, led to marked clinical improvement (7). This could be particularly important early in the disease to prevent or dampen the downstream fibrotic cascade, which is not dependent upon JAK1/JAK2 signaling. However, this will need to be addressed in future clinical studies.

Our study has some limitations. The small sample size of a single individual is due to the extreme rarity of PSM and is further reflected in the sparsity of clinical and molecular data that exist on this disease. However, our study is strengthened by the longitudinal collection of samples, demonstrating the stability of the robust type II IFN–mediated phenotype sustained between the temporally separated biopsies and indicating both the stability of the disease process and the lack of response to treatment. Continual identification and addition of new patients to this scRNA-Seq database will be an important future direction. In addition, the contrast with other fibrotic skin diseases is limited, as we do not have sex- or age-matched patient samples from classic morphea or SSc for comparison, but such a comparison could enhance the characterization of PSM-specific signaling pathways.

In summary, our study describes the inflammatory and fibrotic circuits that exist between T cells, cDC2Bs, and FBs in the skin in patients with PSM and identifies a key inflammatory circuit involving IFN-γ and TGF-β that may be responsible for driving the profibrotic responses and sustaining type II IFN inflammation in PSM skin. Our data support the use of JAK1/JAK2 inhibitors as an early intervention in the management of this disease.

## Methods

### Human sample acquisition.

One PSM and 7 HCs were recruited for scRNA-Seq; the samples from the patient with PSM were also used for spatial sequencing and staining. Biopsies were also taken of 3 patients with morphea and 3 patients with SSc for use in staining.

### scRNA-Seq library preparation, sequencing, and alignment.

Generation of single-cell suspensions for scRNA-Seq was performed as follows. Skin biopsies were incubated overnight in 0.4% dispase (Invitrogen) in HBSS (Thermo Fisher Scientific) at 4°C. Epidermis and dermis were separated. Epidermis was digested in 0.25% Trypsin-EDTA (Thermo Fisher Scientific) with 10 U/mL DNase I (Thermo Fisher Scientific) for 1 hour at 37°C, quenched with FBS (Atlanta Biologicals), and strained through a 70 μM mesh. Dermis was minced, digested in 0.2% Collagenase II (Invitrogen) and 0.2% Collagenase V (MilliporeSigma) in a plain medium for 1.5 hours at 37°C, and strained through a 70 μM mesh. Epidermal and dermal cells were combined in a 1:1 ratio, and libraries were constructed by the University of Michigan Advanced Genomics Core on the 10× Chromium system with chemistry v3. Libraries were then sequenced on the Illumina NovaSeq 6000 sequencer to generate 150 bp paired-end reads. Data processing, including quality control, read alignment (hg38), and gene quantification, was conducted using the 10× Cell Ranger software. The samples were then merged into a single expression matrix using the cellranger aggr pipeline.

### Cell clustering and cell type annotation.

The R package Seurat (v3.1.2) was used to cluster the cells in the merged matrix. Cells with less than 500 transcripts or 100 genes, or more than 1 × 10^5^ transcripts or 15% of mitochondrial expression, were first filtered out as low-quality cells. The NormalizeData function was used to normalize the expression level for each cell with default parameters. The FindVariableFeatures function was used to select variable genes with default parameters. The ScaleData function was used to scale and center the counts in the data set. Principal component analysis (PCA) was performed on the variable genes. The RunHarmony function from the Harmony package was applied to remove the potential batch effect among different batches. The RunUMAP function was used to perform UMAP dimensional reduction. The FindNeighbors and FindClusters functions were used to obtain clusters with the resolution set to 0.6. The FindAllMarkers function was used to find cluster marker genes. The cell types were annotated by overlapping the cluster markers with the canonical cell type signature genes. To calculate the disease composition based on cell type, the number of cells for each cell type from each disease condition was counted. The counts were then divided by the total number of cells for each disease condition and scaled to 100% for each cell type. The FindMarkers function was used to perform differential expression analysis between any 2 groups of cells.

### Cell type subclustering.

Subclustering was performed on the abundant cell types. The same functions described above were used to obtain the subclusters. Subclusters that were defined exclusively by mitochondrial gene expression, indicating low quality, were removed from further analysis. The subtypes were annotated by overlapping the marker genes for the subclusters with the canonical subtype signature genes. The module scores were calculated using the AddModuleScore function on the intended gene lists. The ECM score was calculated on the genes from the extracellular matrix pathway from the Gene Ontology database. The module scores for the upstream regulators were calculated on the target gene lists from the IPA software (Qiagen). To calculate the normalized abundance of disease composition based on cell subtype, the number of cells for each cell subtype from each condition was counted. The counts were then divided by the total cell number for that sample grouping and scaled to 100% for each cell type.

### Integration with FB cytokine signatures.

As previously described by our group (37, 38), we used RNA-Seq–based FB cytokine response signatures for the following cytokines: IFN-γ (5 ng/mL) and TGF-β (10 ng/mL). Primary human FBs from 13 donors were treated with denoted cytokines for 6 hours and harvested for RNA isolation. Unstimulated control FBs were cultured and harvested in parallel. Bulk RNA-Seq was performed on the Illumina NovaSeq 6000 sequencer with the assistance of the University of Michigan Advanced Genomics Core. For each stimulation condition versus unstimulated controls, differential expression analysis was performed using DESeq2 (39). DEGs (2-fold increase; FDR < 0.05) were used to construct response signatures for each cytokine (37). The IFN-γ score for FB subtypes was calculated on induced genes in FBs after stimulation with IFN-γ. The score is the level of IFN-γ response exhibited by each FB subtype, compared to the response signature described immediately prior.

### L-R interaction analysis.

L-R analyses were performed as previously reported. CellPhoneDB (v2.0.0) was applied for L-R analysis. Each subtype was separated by its disease classifications (PSM lesional, nonlesional, or HC), and a separate run was performed for each disease classification. If a subtype contains fewer than 10 cells for a disease classification, it is excluded from this disease classification. Pairs with *P* > 0.05 were filtered out from further analysis. To compare the 2 disease conditions, each pair was assigned to the condition in which it showed the higher interaction score. The number of interactions between each subtype pair was then calculated. The connectome web was plotted using the R package igraph.

### Analysis of CXCL9 levels in the serum of patients with PSM.

We analyzed serum from 9 serum patients with PSM for CXCL9 using the DCX900 Human CXCL9/MIG Quantikine ELISA Kit (R&D Systems). Disease severity was assessed using the LoSAI. Linear regression was used to analyze the correlation between serum CXCL9 concentration and LoSAI score.

### Isolation and culture of FBs from HC skin.

Study participants were recruited from the University of Michigan Scleroderma Program. Dermal FBs were isolated from punch biopsies from the distal forearm of HCs. Negatively selected FBs were cultured in RPMI 1640 with 10% FBS and antibiotics. This study was approved by the University of Michigan IRB, and all participants signed informed consent documents prior to enrollment.

### Cell treatment, RNA extraction, and qPCR.

Dermal FBs from 4 HCs were treated with IFN-γ (5 ng/mL) or TGF-β (10 ng/mL) in RPMI 1640 with 10% FBS and antibiotics for 72 hours. For each condition, cells were then treated with an additional 72 hours of μM of IFN-γ, TGF-β, or both IFN-γ and TGF-β. Gene expression changes in cells were assessed by qPCR after total RNA was extracted using RNeasy Plus Mini Kit (Qiagen, 74134). Reverse transcription was performed using the Applied Biosystems cDNA RT kit (Thermo Fisher Scientific, 4368814), and qPCR was performed with TaqMan Universal PCR Master Mix (Thermo Fisher Scientific) in a 7900HT Fast Real-time PCR system (Applied Biosystems).

### IF staining.

Formalin-fixed paraffin-embedded (FFPE) human tissues for PSM active lesional, PSM lesional center, PSM nonlesional, SSc, morphea, and HC were used, as were frozen slides for PSM lesional, nonlesional, SSc, and HC. A full list of antibodies used can be found in Supplemental Table 1.

FFPE human tissues for PSM active lesional, PSM lesional center, PSM nonlesional, SSc, morphea, and HCs were sectioned and heated at 65°C for 30 minutes, deparaffinized, and rehydrated. Slides were placed in pH 9 antigen retrieval buffer according to manufacturer instructions, heated at 125°C for 30 seconds in a pressure cooker water bath, and then cooled to room temperature. Frozen OCT-embedded tissue was kept at –80°C until sectioning and at –80°C until staining. Frozen slides for PSM lesional, nonlesional, SSc, and HC were incubated at room temperature for 60 minutes and were acetone fixed for 10 minutes. Slides were then washed 3 times for 5 minutes each with phosphate-buffered saline (PBS). FFPE and frozen slides were blocked with 10% serum and incubated with primary rabbit, goat, and/or mouse anti-human antibodies.

Primary antibodies used: anti-Vimentin (rabbit, catalog ab92547, 1.34 μg/mL), anti–IFN-γ (rabbit, catalog ab 9498, 10 μg/mL), anti-CD3 (catalog OriGene UM500048, 8 μg/mL), anti–MHC II (mouse, catalog ab55152 2 μg/mL), anti-CXCL9 (goat, catalog AF392, 20 μg/mL). Appropriate antibodies were coincubated at described concentrations overnight at 4°C. Appropriate negative — no primary or secondary antibodies or isotype control antibodies: rabbit IgG (Abcam ab172730), mouse IgG1 (Abcam ab280974), mouse IgG2b (401201, BioLegend), and goat IgG (AB-108-C, Invitrogen) — antibodies were stained in parallel with each set of the slides mentioned above. Slides were then washed 3 times for 5 minutes each with PBS/Tween 20 (PBST). For secondary antibodies, IFN-γ/CD3 costaining was incubated with tetramethylrhodamine-conjugated (TRITC-conjugated) anti–rabbit IgG (711-025-152, Jackson ImmunoResearch) and Alexa Fluor 488–conjugated anti–mouse IgG (715-545-151, Jackson ImmunoResearch), MHC II/vimentin costaining was incubated with TRITC-conjugated anti–mouse IgG (711-585-152, Jackson ImmunoResearch) and Alexa Fluor 488–conjugated anti–rabbit IgG (711-545-152, Jackson ImmunoResearch), and CXCL9/vimentin costaining was incubated with TRITC-conjugated anti–goat IgG (705-025-147, Jackson ImmunoResearch) and Alexa Fluor 488–conjugated anti–rabbit IgG (711-545-152, Jackson ImmunoResearch). After 30 minutes of coincubation, slides were washed 3 times for 5 minutes each with PBST and were mounted in a Prolong Diamond antifade mountant with DAPI (Invitrogen). Photomicrographs were taken on a Zeiss fluorescence microscope. All IMF exposures were compared against isotype control. The selected frames used in the figures were representative of the whole biopsy.

### IHC staining.

Paraffin-embedded tissue sections (SSc and control skin) were heated at 65°C for 30 minutes, deparaffinized, and rehydrated. Slides were placed in pH 9 antigen retrieval buffer and heated at 125°C for 30 seconds in a pressure cooker water bath. After cooling, slides were treated with 3% H_2_O_2_ (5 minutes) and blocked using 10% serum (30 minutes). Overnight incubation (4°C) was then performed using an anti-human primary antibody. Antibodies used were anti-CLEC10A (6.67 μg/mL, catalog TA810180), anti-CD19 (10 μg/mL, catalog LS-C174739-100), and anti-CD68 (1 μg/mL, catalog ab213363). Slides were then washed and treated with secondary antibody, peroxidase (30 minutes), and diaminobenzidine substrate. Counterstain with hematoxylin and dehydration was done, and slides were mounted and viewed under the microscope.

### Spatial sequencing library preparation and data analysis.

Skin samples were frozen in OCT medium and stored at –80°C until sectioning. Optimization of tissue permeabilization was performed on 20 μm sections using Visium Spatial Tissue Optimization Reagents Kit (10X Genomics), which established an optimal permeabilization time to be 6 minutes. Samples were mounted onto a Gene Expression slide (10X Genomics), fixed in ice-cold methanol, stained with H&E, and scanned under a microscope (Keyence). Tissue permeabilization was performed to release the poly-A mRNA for capture by the poly(dT) primers that are precoated on the slide and include an Illumina TruSeq Read, spatial barcode, and unique molecular identifier (UMI). Visium Spatial Gene Expression Reagent Kit (10X Genomics) was used for reverse transcription to produce spatially barcoded full-length cDNA and for second-strand synthesis followed by denaturation to allow a transfer of the cDNA from the slide into a tube for amplification and library construction. Visium Spatial Single Cell 3′ Gene Expression libraries consisting of Illumina paired-end sequences flanked with P5/P7 were constructed after enzymatic fragmentation, size selection, end repair, A-tailing, adaptor ligation, and PCR. Dual Index Kit TT Set A (10X Genomics) was used to add unique i7 and i5 sample indexes and generate TruSeq Read 1 for sequencing the spatial barcode and UMI and TruSeq Read 2 for sequencing the cDNA insert, respectively. Libraries were then sequenced on the Illumina NovaSeq 6000 sequencer to generate 150 bp paired-end reads.

### Spatial sequencing data analysis.

The reads were aligned to the human genome (hg38), and the expression matrix was extracted using the spaceranger pipeline from 10X Genomics. Seurat was then used for quality control, and Harmony was employed for batch correction. We used conditional autoregressive-based deconvolution (CARD) to provide cell type deconvolution for each spot of the visium data; marker genes are inferred from the scRNA-Seq data, and CARD uses spatial correlation information to infer cell type composition.

### Statistics.

Normality was determined for in vitro data to assess for normal distribution. To determine the differences between groups, 2-tailed unpaired *t* test, Mann–Whitney *U* test, 1-way ANOVA with Sidak test, Kruskal-Wallis test with Dunn’s test, or 2-way ANOVA with Dunnett’s test was performed using GraphPad Prism version 9 (GraphPad Software, Inc). *P* values of less than 0.05 were considered statistically significant. Results were expressed as mean ± SEM unless specified.

### Study approval.

This study involves human participants and was approved by the University of Michigan IRB (HUM00174864). Participants gave informed consent to participate in the study before taking part. This study was conducted according to the Declaration of Helsinki Principles.

### Data availability.

The scRNA-Seq data can be found at Gene Expression Omnibus (accession no. 234987). Values for all data points in graphs are reported in the Supporting Data Values file.

## Author contributions

EX, FM, RW, ACB, LCT, and JEG had access to the study data, developed the figures and tables, and vouch for the data and analyses. EX, FM, RW, and LCT performed the statistical analyses and contributed to data quality control, data analysis, and interpretation of the data. ACB, MGK, JF, CD, AV, MKS, XX, HWC, GB, HJ, PST, RLM, JV, OP, and JMK contributed to data collection, data analysis, and interpretation of the data. JEG and DK directed the work; designed the data collection methods; contributed to data collection, data analysis, and interpretation of the data; and had final responsibility for the decision to submit for publication. All authors contributed intellectual content during the draft and revision of the work and approved the final version to be published. JEG accepts full responsibility for the finished work and/or the conduct of the study, had access to the data, and controlled the decision to publish. JEG and DK contributed equally as last authors. JEG assumes overall responsibility for the content as the guarantor.

## Supplementary Material

Supplemental data

Supporting data values

## Version 1 07/20/2023

In-Press Preview

## Version 2 08/22/2023

Electronic publication
