# Supplementary material for: Pansclerotic morphea is characterized by IFN-γ responses priming dendritic cell fibroblast crosstalk to promote fibrosis
Source: JCI Insight. 2023 Aug 22;8(16):e171307. doi: 10.1172/jci.insight.171307 (PMC10543736; doi:10.1172/jci.insight.171307)
Supplement: Supplemental data [file jciinsight-8-171307-s173.pdf]

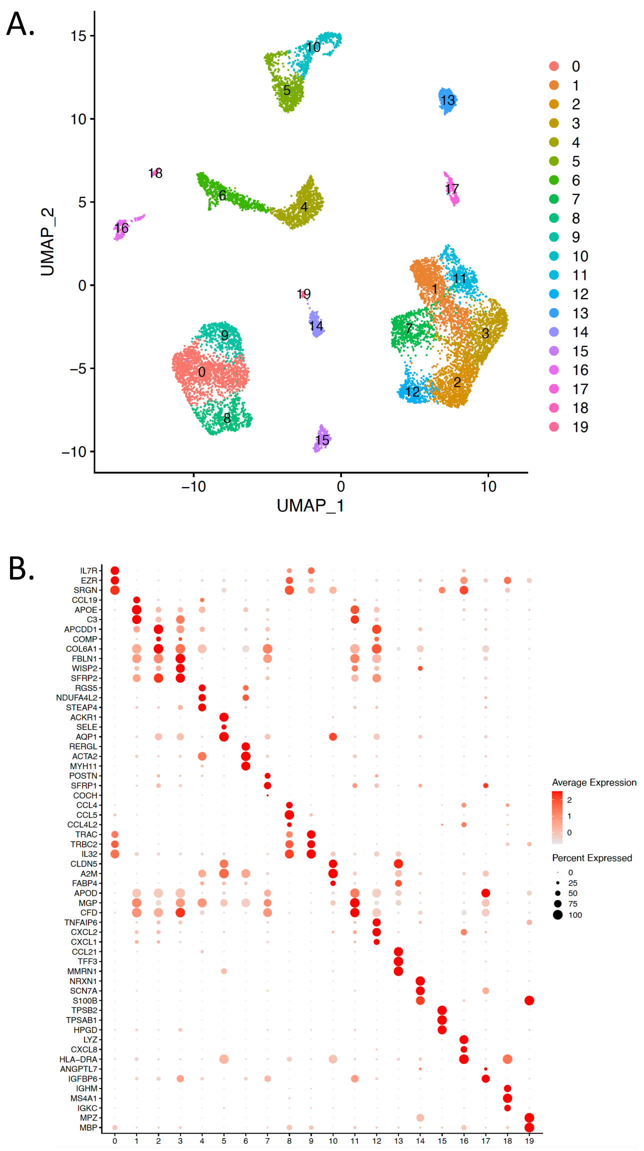

Supplementary figure 1. Pansclerotic morphea dataset clustering. A. UMAP plot of 11,903 cells colored by cluster. B. Dot plot of representative marker genes for each cell cluster.

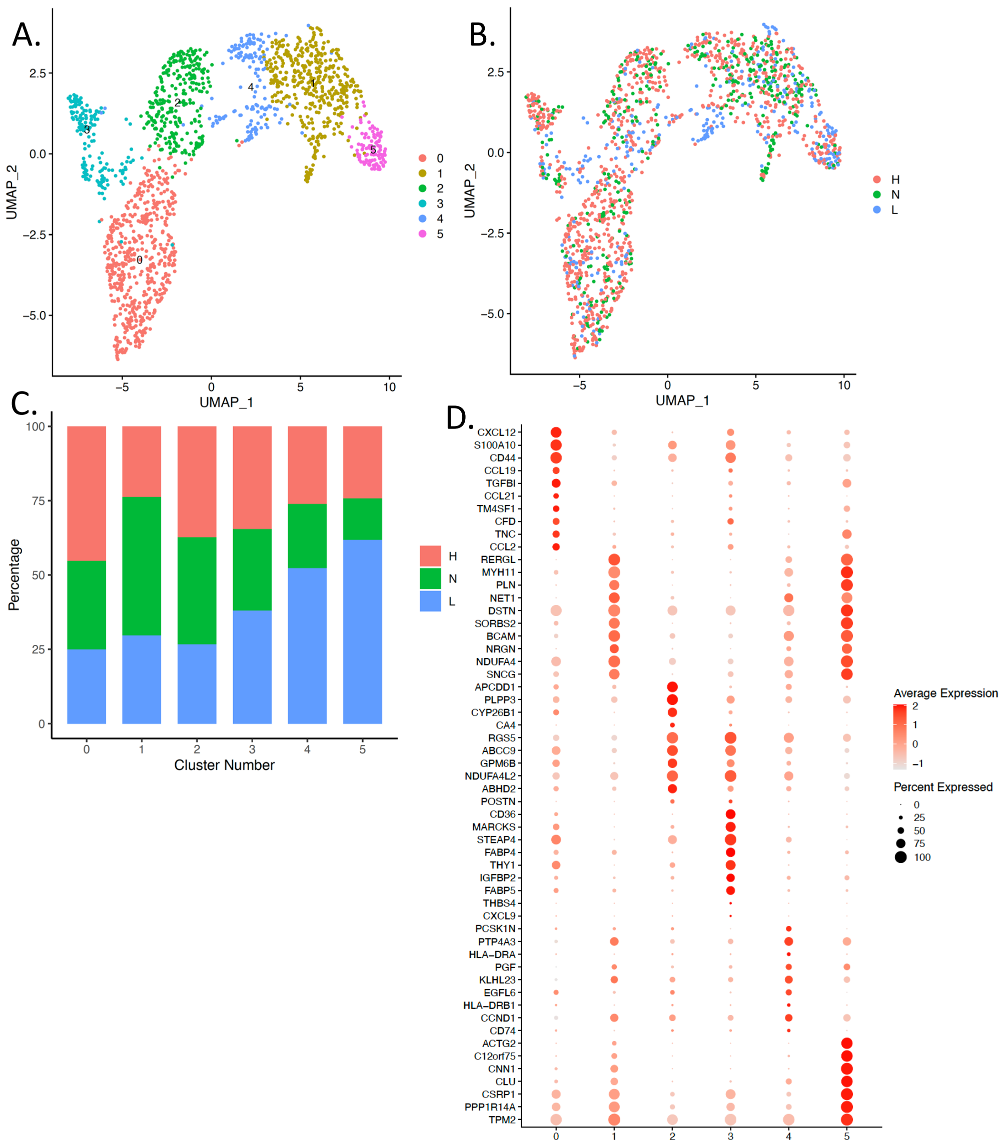

Supplementary figure 2. Pericyte clustering. A. UMAP of 2,694 pericytes colored by subcluster. B. UMAP of pericytes colored by disease state. D. Bar plot showing the relative contribution of the three disease states to the total number of each pericyte subcluster. C. Dot plot of representative marker genes for each pericyte subcluster.

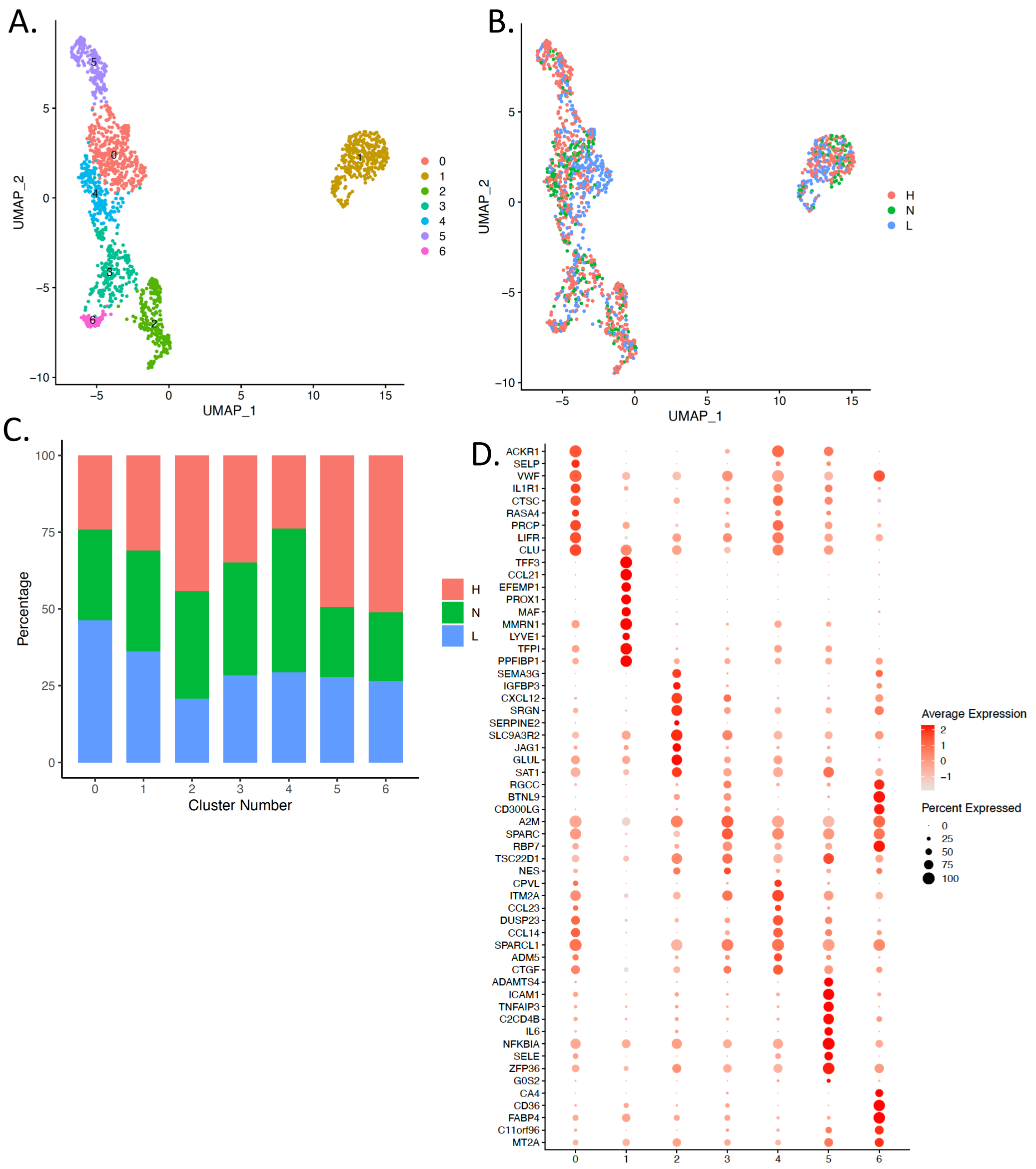

Supplementary figure 3. Endothelial cell clustering. A. UMAP of 1,610 endothelial cells colored by subcluster. B. UMAP of endothelial cells colored by disease state. D. Bar plot showing the relative contribution of the three disease states to the total number of each endothelial cell subcluster. C. Dot plot of representative marker genes for each endothelial cell subcluster.

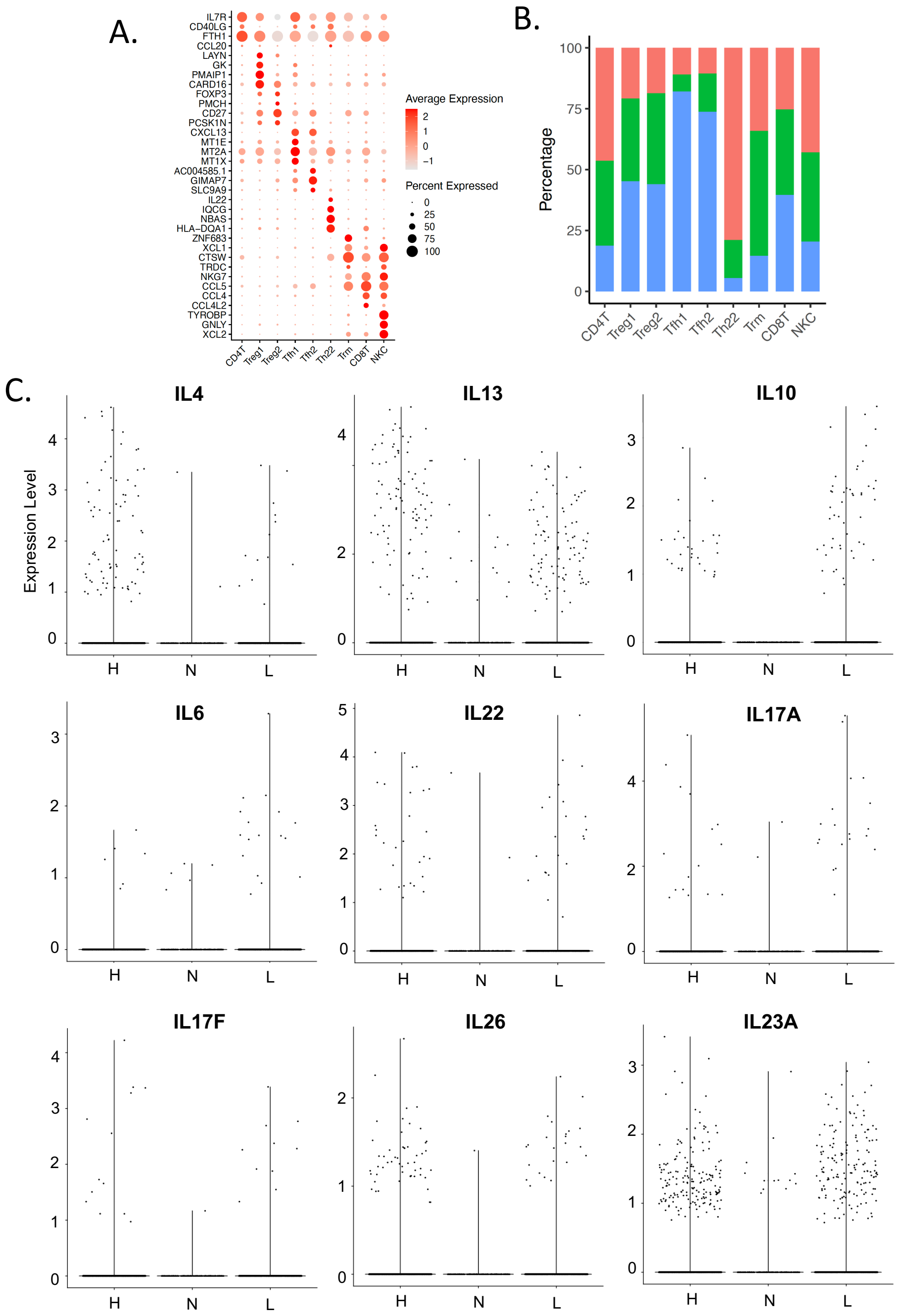

Supplementary figure 4. PSM lesional skin contains enrichment of T cell subtypes. A. Bar plot showing the relative contribution of the three disease states to the total number of each T cell subcluster. B. Dot plot of representative marker genes for each T cell subcluster. C. Violin plots of Th2 and Th17 cytokine expression.

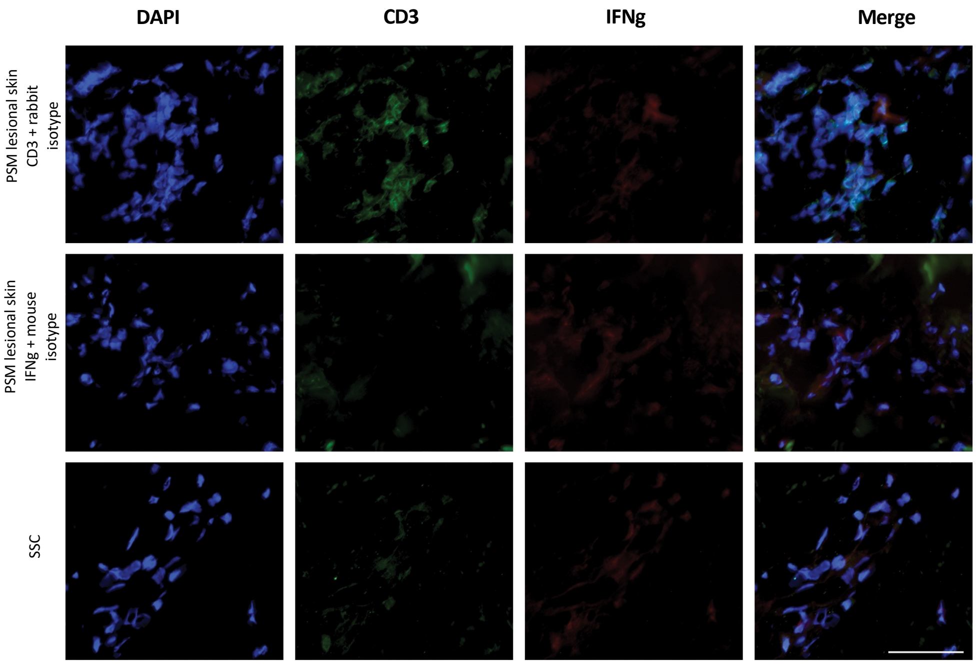

Supplementary figure 5. Isotype controls and SSC co-staining of IFN-γ and CD3

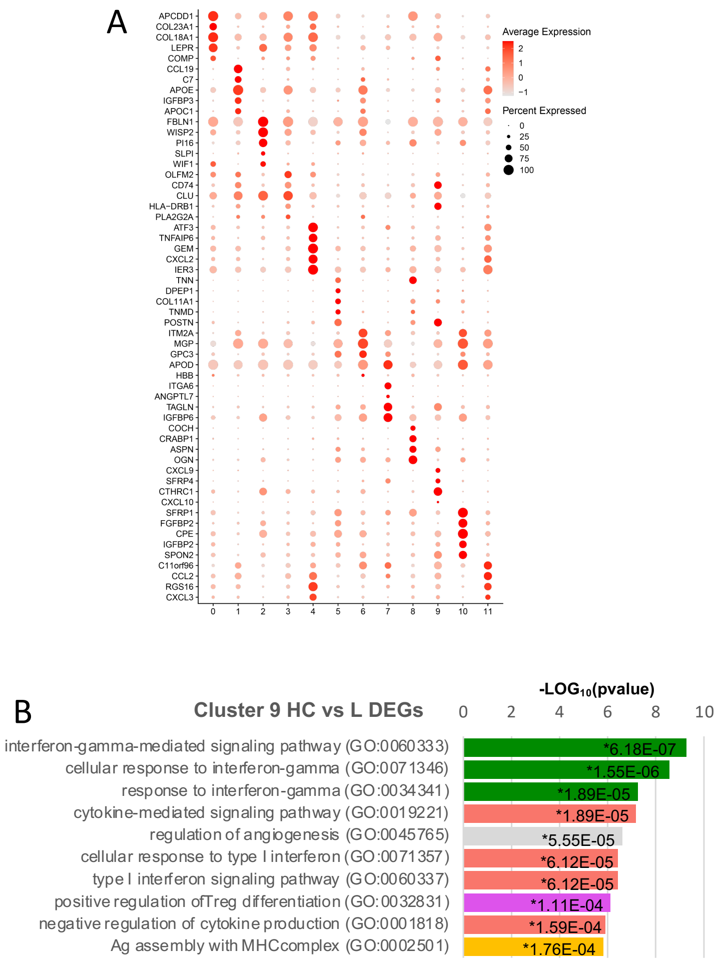

Supplementary figure 6. Fibroblast subclustering and GO analysis. A. Dot plot of representative marker genes for each fibroblast subcluster. B. Gene ontology analysis of upregulated DEGs in lesional PSM vs. healthy control skin in cluster 9 fibroblasts.

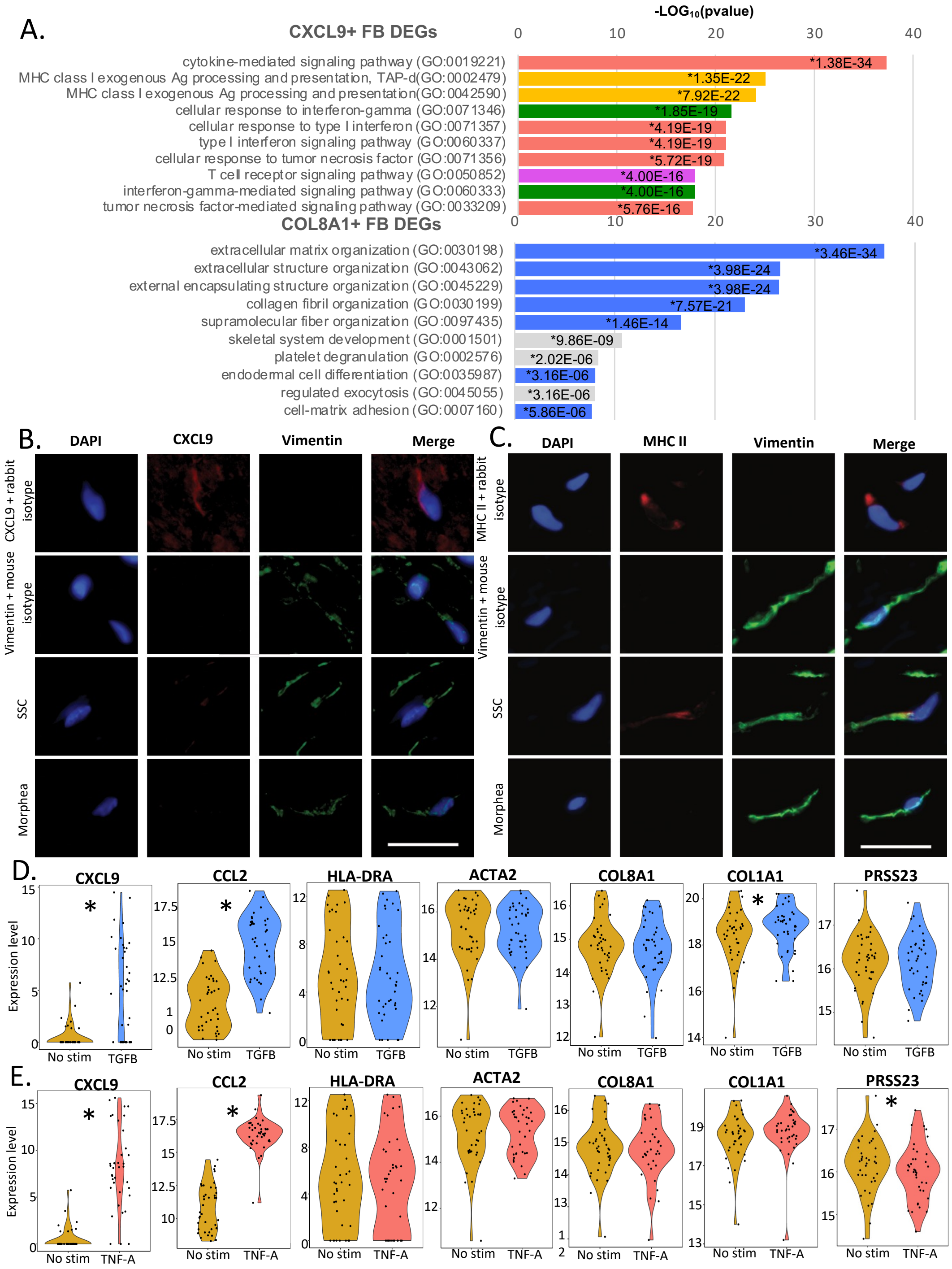

Supplementary figure 7. Differences between *CXCL9+* and *COL8A1+* FBs. *A*. Gene ontology of DEGs significantly upregulated in PSM lesional vs healthy *CXCL9*+ and *COL8A1*+ FBs. Red indicates inflammatory cytokine response, green indicates type II IFN response, yellow indicates antigen presentation, purple indicates T cell receptor signaling, and blue indicates ECM associated pathways. Asterisks are p adjusted values. B, C. Isotype, scleroderma, and morphea staining of *CXCL9*+ vimentin (B), and MHC II + vimentin (C). D, E. Bulk RNA-seq data of healthy fibroblasts after 6 hours TGF-β (D) and TNF (E) stimulation.

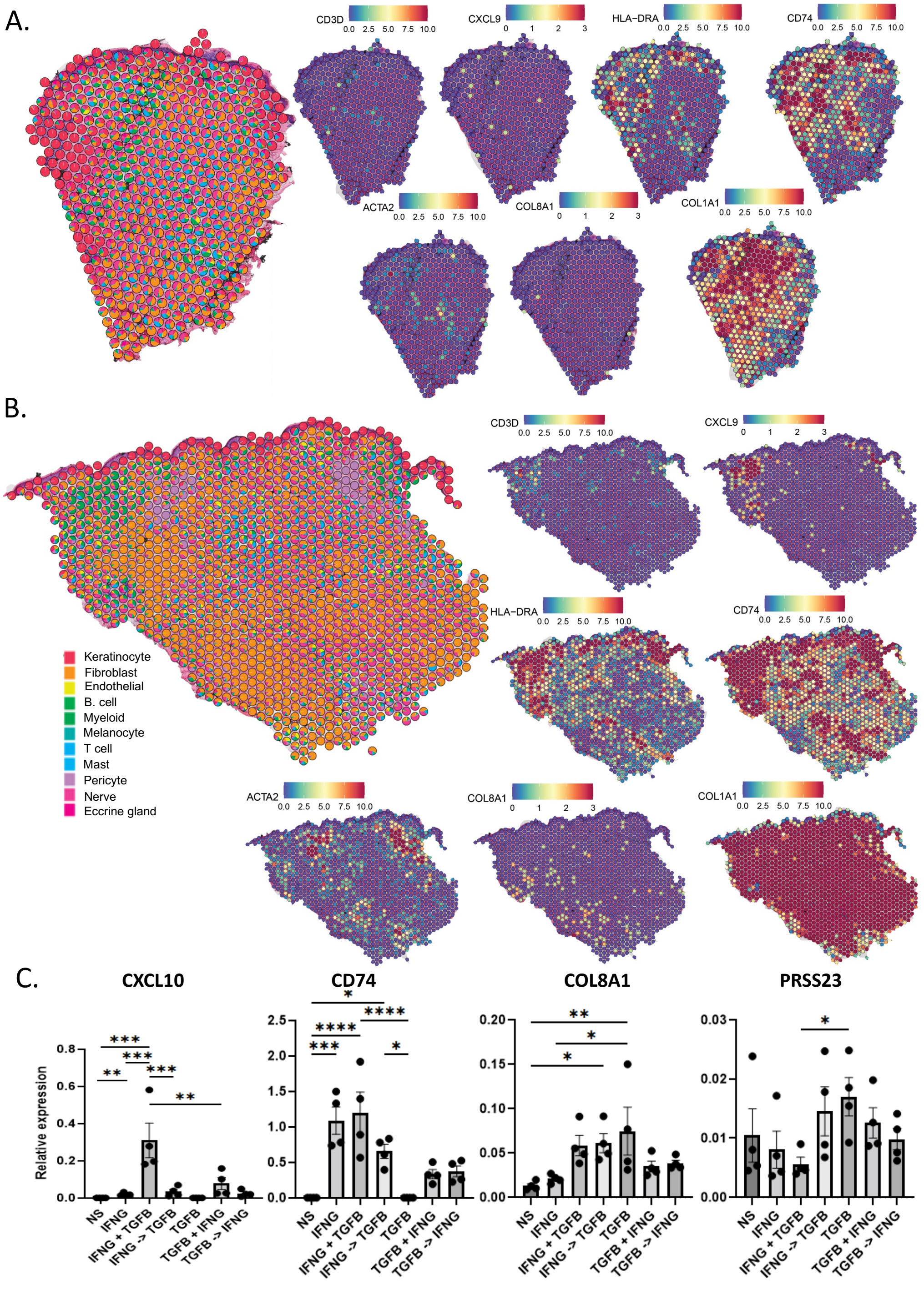

Supplementary figure 8. Lesional and nonlesional gene expression across spatial sequencing, and *in vitro* cytokine stimulation of HC FBs. A. Spatial plot showing deconvoluted cell types overlaid on non-lesional PSM H&E, with coordinates of the spot corresponding to the location in the tissue. Heatmaps indicate *CD3C, CXCL9, HLA-DRA, CD74*, *ACTA2*, *COL8A1,* and *COL1A* expression across the non-lesional spatial plot. B. Spatial plot showing deconvoluted cell types overlaid on lesional PSM H&E. Coordinates of the spot corresponding to the location in the tissue. Heatmaps indicate *CD3C, CXCL9, HLA-DRA, CD74, ACTA2*, *COL8A1,* and *COL1A* expression across the lesional spatial plot. C.

Relative expression of *CXCL10, CD74, COL8A1,* and *PRSS23* after 72-hour priming with IFN-γ or TGF-β, then another 72-hour incubation with the same (IFNG, TGFB), other (IFNG->TGFB, TGFB->IFNG), or both (IFNG+TGFB, TGFB+IFNG) cytokines.

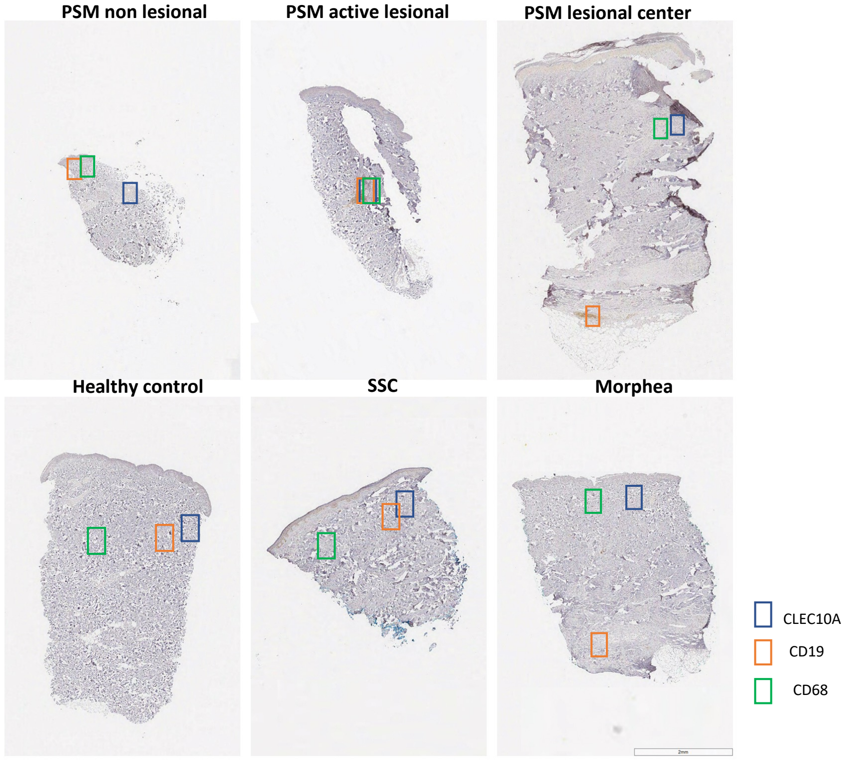

Supplementary figure 9. Locations of IHC staining from PSM nonlesional, active lesional, and lesional center, as well as HC, scleroderma, and morphea skin.

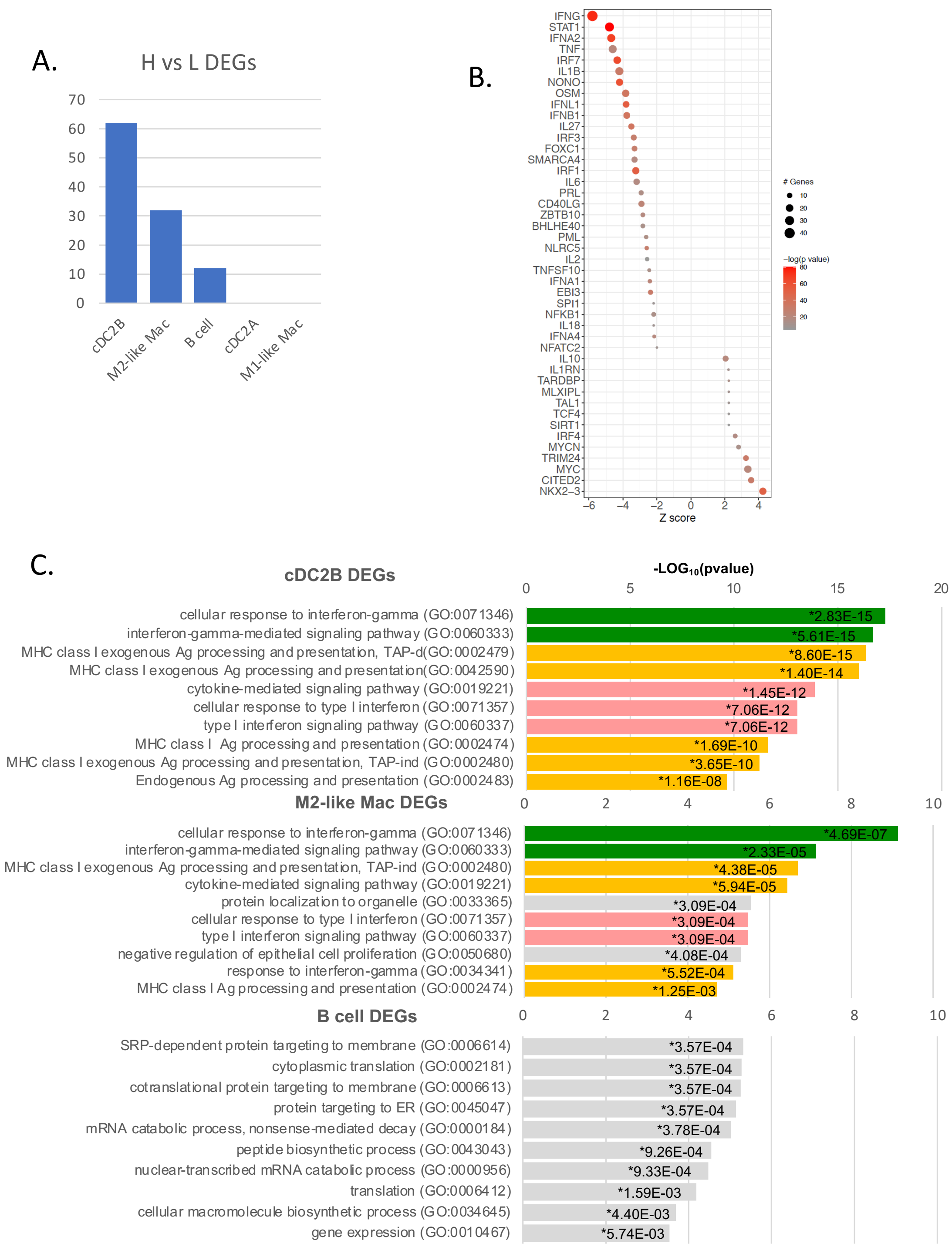

Supplementary figure 10. Healthy vs. lesional PSM myeloid cells. A. Bar graph of the number of DEGs significantly upregulated in PSM lesional vs. HC myeloid cells. No significant DEGs were present in M1-like macrophages. B. Upstream regulator analysis of cDC2B DEGs significantly upregulated in lesional PSM. C. Gene ontology of DEGs significantly upregulated in PSM lesional vs HC myeloid and B cells. Red indicates inflammatory cytokine response, green indicates type II IFN response, yellow indicates antigen presentation, purple indicates T cell receptor signaling, and blue indicates ECM associated pathways. Gray indicates other pathways. Asterisks are p adjusted values.

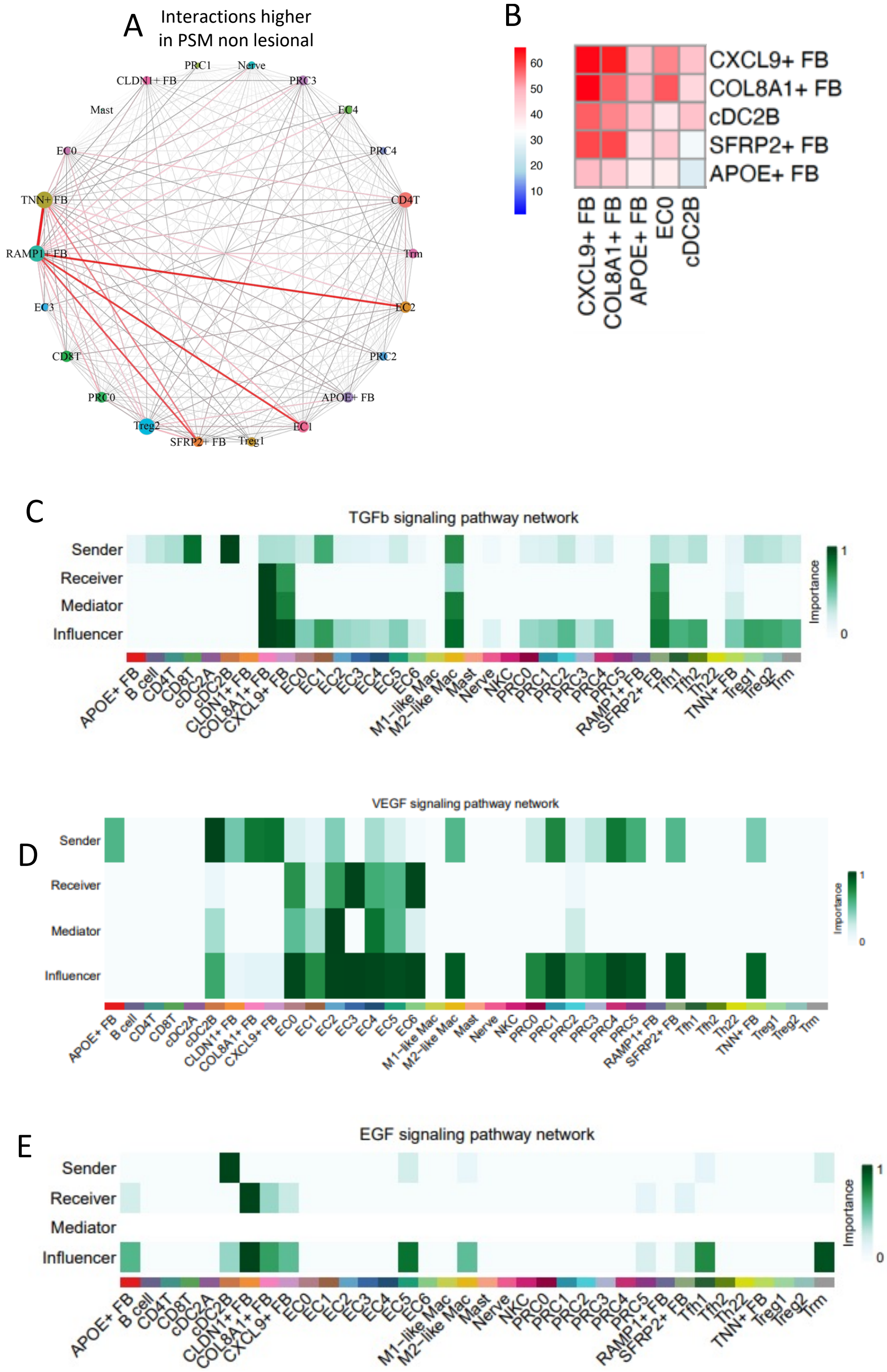

Supplementary figure 11. Cell-cell interactions in PSM skin. A. Connectome web analysis of interacting cell types in PSM nonlesional skin. Line thickness is proportional to the number of interactions between two nodes, and cell type node size is proportional to the number of interactions to and from the cell type. B. Heatmap depicting the number of L-R pairs with interaction scores highest in lesional PSM skin. Row, cell type expressing ligand; column, cell type expressing the corresponding matching receptor. C, D, E. Heatmap showing the relative importance of each expanded cell group based on the computed network centrality measures of the TGF-β (C), VEGF (D) and EGF (E) signaling networks.

Supplementary Table 1

Immunofluorescence		
**Antibody**	**Catalog number**	**Concentration**
anti-Vimentin	ab92547	1.34ug/mL
anti-IFN-γ	ab 9498	10ug/mL
anti-MHC II	ab55152	2ug/mL
anti-CXCL9	AF392, R&D	20ug/mL
anti-CD3	OriGene UM500048	8ug/mL
Rabbit IgG	ab 172730	As needed for primary
Mouse IgG1	ab 280974	As needed for primary
Mouse IgG2b	401201, Biolegend	As needed for primary
Goat IgG	AB-108-C, Invitrogen	As needed for primary
**Secondary Antibody**	**Catalog number**	**Dilution**
TRITC conjugated anti-rabbit IgG	711-025-152, Jackson ImmunoResearch	1/200
Alexa Fluor 488 conjugated anti-mouse IgG	715-545-151, Jackson ImmunoResearch	1/200
TRITC conjugated anti-mouse IgG	711-585-152, Jackson ImmunoResearch	1/200
Alexa Fluor 488 conjugated anti-rabbit IgG	711-545-152, Jackson ImmunoResearch	1/200
TRITC conjugated anti-goat IgG	705-025-147, Jackson ImmunoResearch	1/200
**Immunohistochemistry**		
**Antibody**	**Catalog number**	**Concentration**
anti-CLEC10A	TA810180	6.67ug/mL
anti-CD19	LS-C174739-100	10ug/mL
anti-CD68	ab213363	1ug/mL
